# Per capita sperm metabolism is density dependent

**DOI:** 10.1242/jeb.246674

**Published:** 2024-03-18

**Authors:** Ashley E. Potter, Craig R. White, Dustin J. Marshall

**Affiliations:** Centre for Geometric Biology, School of Biological Sciences, Monash University, Melbourne, VIC 3800, Australia

**Keywords:** Respiratory dilution effect, Sperm respiration, Sperm oxygen consumption, Metabolic plasticity, Metabolic ecology of gametes

## Abstract

From bacteria to metazoans, higher density populations have lower per capita metabolic rates than lower density populations. The negative covariance between population density and metabolic rate is thought to represent a form of adaptive metabolic plasticity. A relationship between density and metabolism was actually first noted 100 years ago, and was focused on spermatozoa; even then, it was postulated that adaptive plasticity drove this pattern. Since then, contemporary studies of sperm metabolism specifically assume that sperm concentration has no effect on metabolism and that sperm metabolic rates show no adaptive plasticity. We did a systematic review to estimate the relationship between sperm aerobic metabolism and sperm concentration, for 198 estimates spanning 49 species, from protostomes to humans from 88 studies. We found strong evidence that per capita metabolic rates are concentration dependent: both within and among species, sperm have lower metabolisms in dense ejaculates, but increase their metabolism when diluted. On average, a 10-fold decrease in sperm concentration increased per capita metabolic rate by 35%. Metabolic plasticity in sperm appears to be an adaptive response, whereby sperm maximize their chances of encountering eggs.

## Teaser

**Summary:** Patterns of density-dependent metabolism extend beyond diploid phases into haploid phases – sperm exhibit metabolic plasticity in response to changes in density, which is likely an adaptive response to the presence of eggs.

## INTRODUCTION

Metabolism is a key driver of ecology and life history as it determines the rate at which organisms use energy and perform biological work (White et al., 2022). Metabolic rate is a highly labile trait – it varies with size, temperature and activity (Gillooly et al., 2001; Glazier, 2008). Although these classic drivers of metabolism are well recognized, it has become apparent that population density also alters metabolic rates. From bacteria to metazoans, organisms at higher densities tend to have lower metabolic rates than conspecifics at lower densities (DeLong et al., 2014; Ghedini et al., 2017; Lovass et al., 2020; Malerba et al., 2017; Marshall et al., 2022). The ultimate driver for this negative association between density and metabolism is unclear, but increased competition for resources is the most commonly proposed explanation (Amundsen et al., 2007; Auer et al., 2015; DeLong et al., 2014). Competition for resources is greater at higher population densities; individuals presumably downregulate their metabolic rates to reduce energy expenditure in the high competition environment (DeLong and Hanson, 2009; DeLong et al., 2014; Malerba et al., 2017). Although these density-metabolism effects are ubiquitous across the tree of life, one group (or gamete type, rather) that has received relatively little attention, despite an august history of the topic, is sperm. Here, we asked: do sperm exhibit density-dependent metabolism, and if so, is the relationship the same as those in metazoans?

Studies of density-dependent regulation of metabolism in multicellular and unicellular organisms have proliferated over recent years (DeLong and Hanson, 2009; DeLong et al., 2014; Ghedini et al., 2017; Lovass et al., 2020; Malerba et al., 2017), but the idea was actually first proposed almost a century ago, and specifically in relation to sperm. In a series of papers starting in the 1920s, Gray (1928a,b,c) showed that sea urchin sperm at high concentrations had lower per capita (individual sperm) metabolic rates and suffered less senescence than sperm in lower concentrations. Gray (1928a,b,c) speculated that the total energy expenditure of the sperm during its life was largely dependent upon dilution (Gray, 1928a). Subsequent studies on the same species confirmed these observations, and the phenomenon was termed the respiratory dilution effect (RDE) (Rothschild, 1948, 1950). These authors alluded to the RDE being adaptive – that sperm are able to minimize their metabolic demands (and prolong their lifespan) when suspended in high concentrations such as those typical of the newly released ejaculate, but increase their activity and metabolism when they become diluted and free swimming (Gray, 1928a). In more modern terminology, the RDE was, in essence, suggested to be a form of adaptive metabolic plasticity: sperm decrease senescence by conserving their finite energy reserves at high densities when the chances of encountering eggs are low, but maximize their competitiveness and fertilization success by increasing their energy consumption at lower densities when the chances of encountering eggs are high.

The RDE emphasizes that sperm metabolism is quite plastic within species; in contrast, sperm competition theory has tended to focus on among-species differences in sperm metabolism and has largely overlooked the potential for intraspecific variation (Boell and Burkus, 1984; Dreanno et al., 1999; Mansour et al., 2003; Rahi et al., 2020). But, as some authors have noted, sperm competition, sperm density and sperm metabolism should be intimately linked (Chia and Bickell, 1983; Reinhardt, 2007; Reinhardt and Otti, 2012). For example, internal fertilizers store sperm from multiple males, maintaining higher sperm concentrations until sperm reach the ova, perhaps maximizing sperm longevity (Reinhardt, 2007; Simmons, 2002). If sperm metabolism is density dependent, then increases in sperm density via the addition of multiple ejaculates could reduce sperm metabolism, a finding that would counter the assumptions of traditional sperm competition theory (Parker, 1993). More generally, the interplay between rates of sperm energy expenditure and their local density remains too poorly understood, but has interesting implications for whether sperm have evolved to outswim or outlive each other (theory makes assumptions about both; Ball and Parker, 1996; Parker, 1993).

Additionally, a range of studies suggest that dilution in female reproductive fluid (i.e. egg secretions or ovarian fluid) can enhance whole-ejaculate sperm metabolism (Gray, 1928b; Hathaway, 1963; Ohtake, 1976), motility (Cornman, 1941; Gasparini et al., 2020; Hadlow et al., 2023; Lillie, 1913; Poli et al., 2019) and longevity (Gasparini et al., 2020; Hadlow et al., 2023; Poli et al., 2019). Whether this reproductive fluid effect is (1) driven by an enhanced resource uptake following dilution, (2) from an adaptive response to egg cues (i.e. chemotaxis) indicating a higher likelihood of encountering eggs or (3) both is unclear.

**Fig. 1.**
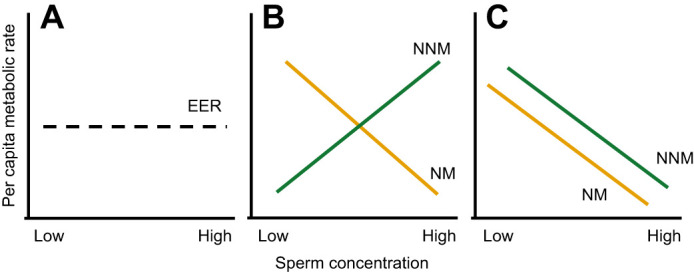
**Schematic showing the potential drivers of metabolic rate in spermatozoa.** (A) No metabolic plasticity. If sperm rely solely on their endogenous energy reserves (EER) and show no plasticity, sperm will have the same per capita metabolism regardless of sperm concentration (black dashed line). (B) Exogenous resources mediate sperm metabolism. If the ejaculate is diluted in non-nutritive media (NNM) (e.g. seawater, carbohydrate-free Ringer’s), sperm in dense suspensions will have higher per capita metabolism because they have access to more resources (green line), whereas when the ejaculate is diluted in nutritive media (NM) (e.g. carbohydrate-containing Ringer’s, seminal fluid), sperm in diluted suspensions will have higher metabolism because they access more resources (yellow line). (C) Adaptive plasticity. If the density–metabolism relationship is driven by adaptive plasticity via cell–cell interaction (e.g. sperm conjugation), sperm in higher concentrations will have lower metabolism regardless of the type of diluent used.

To date, many modern empirical studies in sperm biology explicitly assume that sperm concentration has no effect on per capita sperm metabolic rate (Redenz, 1933) (see *Z*_O_2_ _column in Table S1) (but see references in Chia and Bickell, 1983; Reinhardt, 2007; Reinhardt and Otti, 2012), directly contradicting the RDE hypothesis. So, we have contrasting expectations with regards to whether sperm metabolism depends on density, and if it does, the reasons for this density dependence are also unclear. In Fig. 1, we show alternative hypotheses and how we might distinguish between them. If dilution with carbohydrate-containing media increases per capita metabolic rate but dilution with carbohydrate-free media does not, then we can infer that resource limitation is driving density-dependent sperm metabolism. Otherwise, if increases occur regardless of diluent type, then plasticity in response to local biochemical conditions mediated by sperm density (e.g. carbon dioxide, bicarbonate, pH; Grahn et al., 2023), cell–cell interactions (e.g. quorum sensing; Luther and Waberski, 2019) or sperm conjugation (Higginson and Pitnick, 2011; Monclus and Fornes, 2016) could drive the effect.

To resolve the uncertainty surrounding the relationship between sperm metabolism and density, we conducted a comparative analysis. We estimated how sperm metabolism covaries with density among and within species and gained insight into the drivers of these patterns. Our analysis included 198 measures of sperm metabolism from 49 species across five phyla, encompassing a wide range of sperm concentrations (∼6 orders of magnitude). We categorized species into endotherms and ectotherms to account for the effect of thermoregulation on sperm metabolism because thermoregulation is a strong predictor of metabolic rate more generally (Glazier, 2010). We also categorized diluents as either carbohydrate-free or carbohydrate-containing media to explore the effect of resources on density-dependent metabolism. We estimated the covariance between aerobic metabolism and sperm density (including either thermoregulation or diluent type) using a linear-mixed model framework. Our estimate of metabolism focused solely on aerobic metabolism, as it was the most commonly reported metric, but we acknowledge that aerobic and anaerobic metabolism are highly interdependent metabolic processes (Ruiz-Pesini et al., 2007). Lastly, we assessed the degree of misestimation when it was assumed that sperm metabolism was independent of density.

## MATERIALS AND METHODS

### Comparative analysis of sperm metabolism

#### Protocol, registration and reporting

We followed the PRISMA (Preferred Reporting Items for Systematic reviews & Meta-Analyses; Moher et al., 2016; O'Dea et al., 2021) approach to help build our database (PRISMA Checklist; Table S2), but we used more traditional comparative analyses to test our hypothesis – a hybrid between a meta-analysis and a comparative analysis. This hybrid approach allows us to use the formality of meta-analytic search techniques but also use standard metabolic scaling approach for the analysis (see Supplementary Materials and Methods, Data extraction and effect size). This is a standard technique that has been used previously (Barneche et al., 2018; Marshall et al., 2020; Pettersen et al., 2019) but it means that we cannot include all of the steps of a formal meta-analysis.

#### Literature searches and study selection

We aimed to collect a comprehensive dataset using published and unpublished data to understand the relationship between sperm metabolism and sperm density across species. Studies were collected from ISI Web of Science (http://www.webofknowledge.com/WOS) and Google Scholar (https://scholar.google.com/) from 2019 to 2023. We used specific search strings based on the database (see Supplementary Materials and Methods). Studies cited within searched studies were also included in our dataset if they met the criteria. Our search strategy involved: (1) inputting the search terms into our information sources; (2) assessing titles for relevance; (3) if relevant, downloading the title, author and abstract and uploading to Rayyan QCRI (Ouzzani et al., 2016); (4) reading the abstract for relevance [(a) in title: includes sperm OR spermatozoa OR gametes AND some form of activity, energy use OR sperm quality and (b) in abstract: includes sperm OR spermatozoa OR gametes AND metabolism, respiration, oxygen consumption rate, mitochondrial respiration, energy use, sperm quality OR sperm activity]; (5) reviewing the entire paper for relevance based on criteria (Table S3); and (6) extracting the data. If the studies did not meet the criteria presented in Table S3, they were excluded from the dataset. For further information on eligibility criteria, see Supplementary Materials and Methods (Eligibility criteria) and Fig. S1. Although we attempted to collect all relevant data on sperm metabolism, we recognize that some important metabolic parameters [i.e. FAD, NAD(P)H, ATP production] and studies (e.g. Massino et al., 2021) could not be included. We used oxygen consumption rate as our measure of metabolism because it is the most widely used metabolic parameter for measuring metabolism and allowed us to include the greatest number of species and studies, increasing the reliability and generality of our findings. Overall, our dataset included 198 observations of aerobic metabolism from 88 studies conducted from 1950 to 2022, including 49 species (21 endotherms and 28 ectotherms).

#### Data extraction and effect size

Our data was primarily collected from original sources because there were few compilations (Jones and Murdoch, 1996) that provided information on metabolism in sperm for a range of species that were also explicit with regards to concentration. We used raw or averaged data from original sources which were extracted from tables or figures using Webplotdigitizer (v. 4.5; Rohatgi, 2020). Papers cited in these original sources were also investigated and included in our database if they followed the criteria presented in Table S3. We also included data for two species collected by the authors (see Supplemental Materials and Methods, Empirical estimates). Species were categorized by thermoregulation (endotherm and ectotherm) which largely covaries with fertilization mode in our dataset [i.e. internal=endotherms, external=ectotherms (except for *Apis mellifera* and *Loligo pealei*, both ectotherms with internal fertilization)] (Fig. S2). We also recorded information on the diluent used to dilute the sperm. Diluents were categorized as either carbohydrate-free (i.e. containing no sugar) or carbohydrate-containing (i.e. containing sugar) media. Last, we reported sperm handling (i.e. fresh, cooled or frozen–thawed), extraction method [i.e. ejaculated, extracted (male) or extracted (female)] and diluent type (i.e. activation or extender) for each species/reference in the dataset (Table S1).

We did not scale our data by their error to calculate effect size because doing so would remove the quantitative relationship that we are interested in (see Supplementary Materials and Methods, Data extraction and effect size). However, we did quantify the effect of concentration on per capita sperm metabolism by limiting our dataset to four studies that reported ‘high’ and ‘low’ sperm concentrations (∼10-fold difference) and calculated a ln-transformed response ratio (LRR) (Borenstein et al., 2009). The LRR was calculated as: LRR=ln(*X*_L_/*X*_H_), where *X*_L_ is the mean per capita sperm metabolism at lower concentrations and *X*_H_ is the mean per capita sperm metabolism at higher concentrations. We ran a model with log response ratio as our response variable. Our results show a significant positive effect of concentration on per capita metabolic rate (*F*_1,4_=15.6, *P*=0.017): per capita sperm metabolism was 1.2-fold higher at lower sperm concentrations. We also ran a model where we excluded studies with lower replication (<4 replicates) and found no qualitative differences in our results to those that included the entire dataset (see Results).

#### Empirical estimates

To expand the number of species and data points in our study, we included data that we had collected ourselves. We estimated density-dependent metabolism in two broadcast spawning invertebrates, *Galeolaria caespitosa* (a calcareous tube worm) and *Heliocidaris erythrogramma* (a sea urchin)*.* Colonies of *G. caespitosa* were collected from the St Kilda pier (Melbourne, Australia; 37°51′53.964″S, 144°58′8.256″E), and placed in coolers filled with seawater and transported to Monash University. Spawning was induced using a standard method specifically for *G. caespitosa* (Marshall and Evans, 2005). The ejaculate was collected immediately after spawning using a 1.5 ml syringe, pooled together and diluted to several concentrations (10^6^–10^8^ sperm ml^−1^) using 0.2 µm filtered seawater. Sperm density was estimated for each sperm concentration using a haemocytometer (4 replicates per dilution).

*Heliocidaris erythrogramma* was collected near Ricketts Point (Melbourne, Australia: 37°59′26.016″S, 145°1′39.468″E) and transported in coolers back to Monash University. Spawning was induced by injecting 5 ml of 0.5 mol l^−1^ KCl into the peristomal membrane and then placing each sea urchin over a beaker to collect the ejaculate. A single male ejaculate was diluted to several concentrations (10^5^–10^7^ sperm ml^−1^) using filtered seawater and sperm density was estimated as mentioned above.

Sperm metabolic rate was measured as oxygen consumption over time in an air-tight chamber. We used several 24-channel PreSens sensor dish readers (Sensor Dish Reader SDR2, PreSens, Germany) with 24-chamber 750 µl glass microplates to measure oxygen consumption over time (% O_2_ h^−1^) (White et al., 2011), where measurements were taken every minute for 30–60 min, depending on the concentration. The 750 µl glass chambers contained non-consumptive O_2_ sensor spots that were calibrated with air-saturated seawater (100% AS) and water containing 2% sodium sulfide (0% AS) prior to measurements. Sperm concentration was randomized across the 24-chamber microplates and replicated 4–6 times, while the remaining four chambers were kept free of sperm and contained only filtered seawater as a control. Oxygen measurements were recorded in the dark at a constant temperature of 21°C (ambient ocean temperature), with the plates flipped on their sides to prevent the sperm from settling directly onto the sensor spot, located at the bottom of each vial. Oxygen consumption (% O_2_ h^−1^) was converted to metabolic rate (*V̇*_O_2__, μl O_2_ h^−1^; LoLinR package; Olito et al., 2017) using the rate of change of oxygen saturation for controls vials, oxygen capacitance of air-saturated seawater at 21°C (5.11 ml l^−1^) (Cameron, 1986) and volume of water in the chamber (750 µl) (White et al., 2011). The rate of change was taken within 100–75% oxygen saturation for *G. caespitosa* for all concentrations (10^6^–10^8^ sperm ml^−1^). However, for *H. erythrogramma*, oxygen declined rapidly with higher concentration such that oxygen saturation started at ∼60% for higher concentrations (10^7^ sperm ml^−1^) but started at 100% for lower concentrations (10^5^−10^6^ sperm ml^−1^), so we only used 10^5^−10^6^ sperm ml^−1^ in the analyses to ensure our estimates of metabolism were compared across similar oxygen saturation ranges.

### Misestimation

Papers typically reported metabolism as a standardized rate (i.e. *Z*_O_2__=µl O_2_ 10^8^ sperm ml^−1^ h^−1^) meaning that they transformed the metabolic rate to what it would be based on a standardized sperm concentration (i.e. 10^8^ or 10^9^ sperm ml^−1^) (Redenz, 1933) instead of reporting the rate that the actual sperm concentration respiration was measured at (i.e. 3.2×10^8^ sperm ml^−1^). To report sperm metabolism at a standardized concentration, most studies [67% or *n*=62 (species=38)] used a linear transformation to extrapolate metabolic rates from the concentration under which it was measured to the standardized value: this approach explicitly assumes that sperm metabolic rate is unaffected by sperm density. Hence, using a linear transformation introduces systematic biases if the relationship between sperm concentration and sperm metabolism is actually nonlinear (which it is; see Results). For example, when a study reported a standardised metabolic rate of 1.34 µl O_2_ 10^8^ sperm ml^−1^ h^−1^ but the methods reported that the actual sperm concentration used during the metabolism measurement was 3×10^8^ sperm ml^−1^, we were able to back-calculate the estimate to get to the actual metabolic rate that was measured. To back-calculate, we first divided the reported metabolic rate provided (1.34 µl O_2_ h^−1^) by the standardised sperm concentration (10^8^ sperm ml^−1^), then multiplied that value by the actual sperm density reported in the methods of the paper (1.34×10^−8 ^µl O_2_ h^−1^×3×10^8^ sperm ml^−1^=4.02 µl O_2_ h^−1^ for 3×10^8^ sperm ml^−1^). This approach represents the same linear transformation as was used by the original author (Redenz, 1933). Data from other papers [28% or *n*=31 (species=33)] did not need to be transformed because metabolic rate was reported at the actual sperm density that was used. A subset of papers [*n*=20 (species=16)] could not be included in the analyses because they did not report the actual sperm density and only reported a standardized metabolic rate.

We wanted to understand the extent to which papers overestimated or underestimated sperm metabolism, so we calculated the percent misestimation for papers that reported standardized sperm metabolism, using the following equation:
(1)

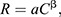


where *R* is the metabolic rate, *a* is the coefficient, *C* is the sperm concentration and β is the density–metabolism exponent for sperm. First, we rearranged Eqn 1 to solve for *a*:
(2)

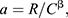


where we used the actual sperm concentration used in the experiment (*C*), actual metabolic rate (*R*) and the density–metabolism exponent found for sperm across species (β=0.87; see Results). Then we used *a* from Eqn 2 and plugged it into Eqn 1 with the standardized sperm concentration as *C*, to find the ‘actual’ metabolic rate, i.e. metabolic rate that was supposed to be measured if the standardized concentration was actually used. Next, we used the ‘actual’ metabolic rate to find the percent misestimation using this equation:
(3)

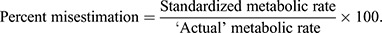


Finally, we calculated the fold change (i.e. increase or decrease) in sperm concentration using the actual sperm concentration used in the experiment and the standardized reported sperm concentration:
(4)

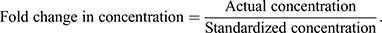


### Statistical analyses

All analyses were done in R (v. 4.2.2, https://www.r-project.org/), and the assumptions of a linear model were checked using Q-Q plots, histograms and boxplots of residuals. We constructed a tree from the Open Tree of Life (OTL) using the rotl R package to account for phylogenetic non-independence (Michonneau et al., 2016). We first explored our data using a series of phylogenetically controlled linear mixed-effects (random intercept) models with species as a random effect and sperm concentration and thermoregulation (two levels: endotherm, ectotherm) or diluent (two levels: carbohydrate-containing, carbohydrate-rich) as fixed effects using the phyr::pglmm R package (Ives, 2018). Thermoregulation was included as a factor because it is a strong predictor of metabolic rate (Glazier, 2010). When we compared models with and without phylogeny, we found no difference in model fit (χ^2^=0.15, *P*=0.70, ΔAIC<2) and the results were quantitatively and qualitatively similar. Therefore, we decided to use a linear mixed-effects model (using the lmer R package) with species as a random effect. We used a multistep approach for our statistical analyses where we evaluated the overall significance of full model effects using analysis of deviance tests based on χ^2^ distributions and Akaike’s information criterion (AIC; Akaike, 1973) and then reduced models in which interactions were not significant (*P*>0.05). We then used a Wald test to determine whether the coefficient was significantly different from 1. We combined both empirical observations and data compiled from external sources for this analysis.

## RESULTS

### Comparative analysis of sperm metabolism

**Fig. 2.**
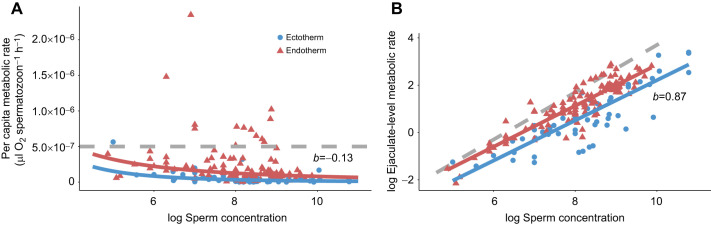
**Among-species relationships between density-dependent per capita and ejaculate-level metabolic rates.** Fitted lines represent line of best fit based on a linear mixed-effects model using all the data in the dataset. (A) Relationship between per capita metabolic rate (µl O_2_ spermatozoon^−1^ h^−1^) and log_10_ sperm concentration (sperm ml^−1^) (*b*=−0.13). The *y*-axis is on a non-log scale for ease of view. The two outliers did not change the density–metabolism relationship, so they were left in. (B) Relationship between log_10_ ejaculate-level metabolic rate (µl O_2_ h^−1^) and log_10_ sperm concentration (*b*=0.87). Lines within plots do not significantly differ from each other. Each point represents an observation from a single species for 49 species total (endotherms: red triangles, *n*=123; ectotherms: blue circles, *n*=75). Grey dotted lines represent a linear relationship (or no density-dependence) between sperm metabolism and concentration. Data were analysed in a natural log framework but are displayed on a log_10_ scale for ease of view.

**Fig. 3.**
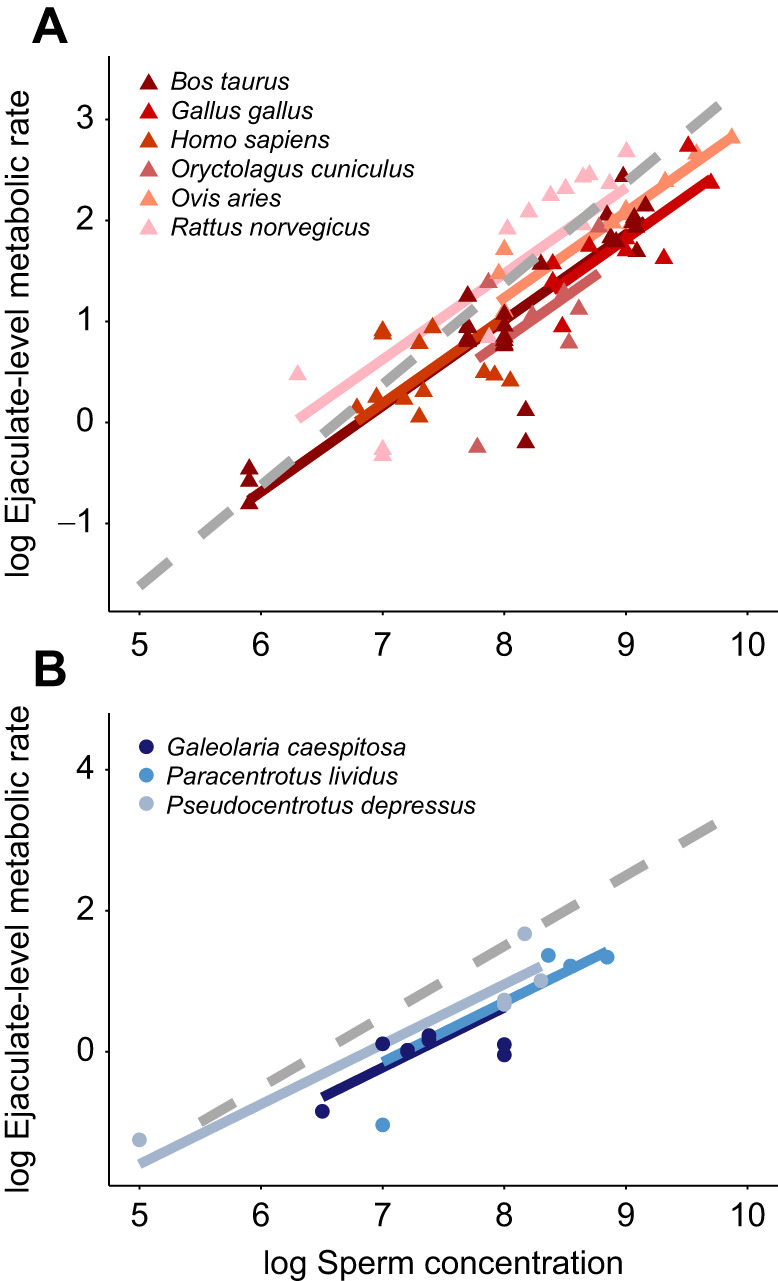
**Within-species relationships between sperm concentration and ejaculate-level metabolic rate.** Fitted lines represent line of best fit based on a phylogenetic linear mixed-effects model using a limited dataset for species for which we had multiple measures of sperm concentration (nine species). Plots show the relationship between ejaculate-level metabolic rate (µl O_2_ h^−1^) and sperm concentration on log_10_–1og_10_ axes for (A) endotherms and (B) ectotherms. Each data point represents a single observation of metabolic rate for a given species. Slopes did not differ among species, so we fit the same slope to each species (*b*=0.87). Dotted lines show the expected relationship if density (concentration)-dependent metabolic plasticity was not observed (*b*=1). Data were analysed in a natural log framework but are displayed on a log_10_ scale for ease of view. Data for *Galeolaria caespitosa* were collected empirically and the remaining species were collected from other sources. (Note: 9 out of the 12 species were plotted for ease of view.)

We found that the relationship between sperm concentration and sperm metabolism is nonlinear – sperm have lower per capita (i.e. individual) metabolisms at higher densities relative to sperm at lower densities (Fig. 2). In more formal statistical terms, the scaling exponent for per capita metabolism and concentration is −0.13 (Fig. 2A), meaning that the scaling exponent for ejaculate-level (i.e. sample of multiple sperm) metabolism and concentration is 0.87 (Table 1), which was significantly different from 1 (Wald test, *t*_198_*=*3.25, *P=*0.001; Fig. 2B). Additionally, there was no evidence that the relationship between sperm concentration and metabolism differed significantly within different species (Fig. 3): for 11 species for which we have multiple measures of metabolism across a range of concentrations (∼100-fold difference), the species×concentration interaction was not significant (ΔAIC=0.3, *P*=0.12). However, this nonsignificant interaction needs to be interpreted with caution considering that the sample size for most species was small (*n*=4 to 28).

**Table 1.**
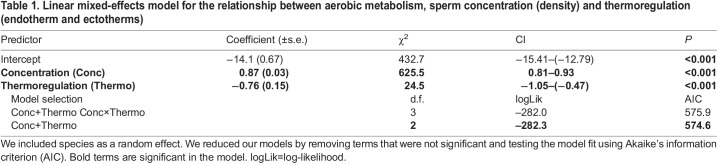
Linear mixed-effects model for the relationship between aerobic metabolism, sperm concentration (density) and thermoregulation (endotherm and ectotherms)

We found that density effects were similar among endotherms and ectotherms but there was a significant difference in the oxygen consumption rate between sperm of endotherms and ectotherms (Table 1): sperm of endotherms have ∼2-fold higher metabolisms than sperm of ectotherms (Fig. 2). The results did not change when we excluded studies with lower replication (<4 replicates) (χ^2^_Thermoregulation×Concentration_=0.02, d.f.=1, *P*=0.69; χ^2^_Concentration=_429.4, d.f.=1, *P*=<0.001, coefficient±s.e.=0.83±0.04; χ^2^_Thermoregulation=_10.1, d.f.=1, *P*=0.001). As endotherms were exclusively internal fertilizers and all but two of the ectotherms were external fertilizers, disentangling endothermy effects from fertilization mode effects is not possible (see Fig. S2). We found that density effects were similar among fertilization modes (χ^2^_Fertilization mode×Concentration_=0.72, d.f.=1, *P*=0.40) and our main result remained the same (χ^2^_Concentration_=620.8, d.f.=1, *P*=<0.001, coefficient±s.e.=0.86±0.03).

**Fig. 4.**
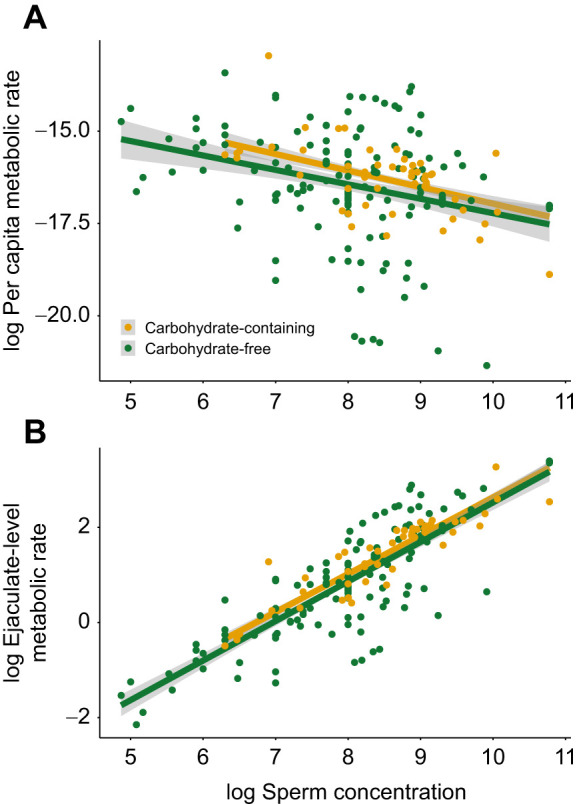
**Among-species relationships between density-dependent per capita and ejaculate-level metabolic rates based on the diluent.** Plot shows the relationship between (A) per capita metabolic rate and sperm concentration (sperm ml^−1^) and (B) ejaculate-level metabolic rate (µl O_2_ h^−1^) and sperm concentration (sperm ml^−1^) on log_10_–log_10_ axes separated by diluent (i.e. carbohydrate-containing and carbohydrate-free). Fitted lines and confidence intervals represent line of best fit based on a linear mixed-effects model. Each point represents an observation from a single species for 49 species. Colours indicate different diluents (green, carbohydrate-free media, *N*=148; yellow, carbohydrate-containing media, *N*=50; see Supplementary Materials and Methods for more information on diluent classification).

We compared the density-dependent metabolism of sperm diluted with different media types (i.e. carbohydrate-containing versus carbohydrate-free) and found no significant interaction or main effect of diluent (Table 2) (Fig. 4): sperm experienced the same decline in per capita density-dependent metabolism regardless of the diluent used (Fig. 4A). We also analysed the effects of methodological differences in sperm handling (i.e. storing, extraction, diluent) and found that none of these methods altered the relationship between density and metabolism (Table S1). We reduced our dataset to only freshly collected sperm (excluding frozen–thawed or cooled sperm) and found that our results remained unchanged (χ^2^_Thermoregulation×Concentration_=1.98, d.f.=1, *P*=0.16; χ^2^_Concentration_=492.2, d.f.=1, *P*=<0.001, coefficient±s.e.=0.87±0.04; χ^2^_Thermoregulation=_25.3, d.f.=1, *P*=<0.001). We also analysed the density-dependent effects of different extraction methods (i.e. ejaculated or extracted from female) on sperm metabolism and found no significant difference (χ^2^_Extraction method×Concentration_=0.59, d.f.=1, *P*=0.44). Similarly, diluent job (i.e. activating or extending) had no effect on density-dependent metabolism of sperm (χ^2^_Diluent Job×Concentration_=1.40, d.f.=1, *P*=0.24) but the use of an extender diluent did increase oxygen consumption slightly (χ^2^_Diluent Job=_6.86, d.f.=1, *P*=0.01).

**Table 2.**
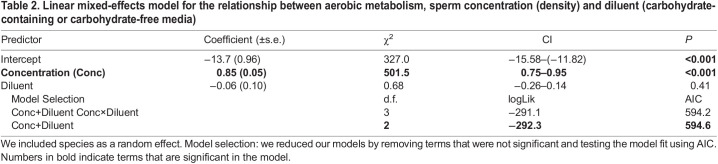
Linear mixed-effects model for the relationship between aerobic metabolism, sperm concentration (density) and diluent (carbohydrate-containing or carbohydrate-free media)

### Misestimation

**Fig. 5.**
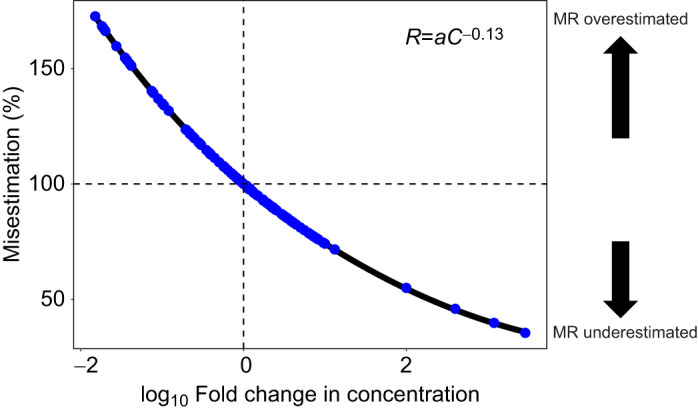
**Percent misestimation of sperm metabolism based on the fold change in reported sperm concentration relative to the actual sperm concentration.** Misestimation was calculated as the proportion of the adjusted standardized sperm metabolism (adjusted for the actual scaling relationship, *b=*0.87) and the standardized sperm metabolism reported from literature. Sperm concentration ratio was calculated as the proportion of the actual sperm concentration and the standardized sperm concentration on a log scale. Misestimation was calculated using Eqn 1, where *R* is the respiration rate, *a* is the coefficient and *C* is the concentration. Dashed lines represent the boundaries for misestimation: the point of intersection, no misestimation; right of vertical line, standard sperm concentration is less than the actual sperm concentration; left of vertical line, standard sperm concentration is greater than the actual sperm concentration; above horizontal line, sperm metabolic rate is overestimated; below the horizontal line, sperm metabolic rate is underestimated.

We found that assuming a linear relationship between sperm concentration and metabolism introduced systematic misestimates of sperm metabolic rate. The direction and magnitude of the misestimation depended on the concentration (*C*) at which respiration was measured relative to the standardized concentration reported. When the actual sperm concentration was lower than the standardized concentration, linear transformations systematically overestimated metabolic rate at the standardized concentration (Fig. 5). When the actual sperm concentration was higher than the standardized concentration, linear transformations systematically underestimated metabolism at the standardized concentration. The relationship between the degree of misestimation follows the form of *y=x*^−0.13^, where *y* is the degree of misestimation (%) and *x* is the log-fold difference between the actual sperm concentration used in the experiment and the standardized reported sperm concentration (Fig. 5).

## DISCUSSION

**Fig. 6.**
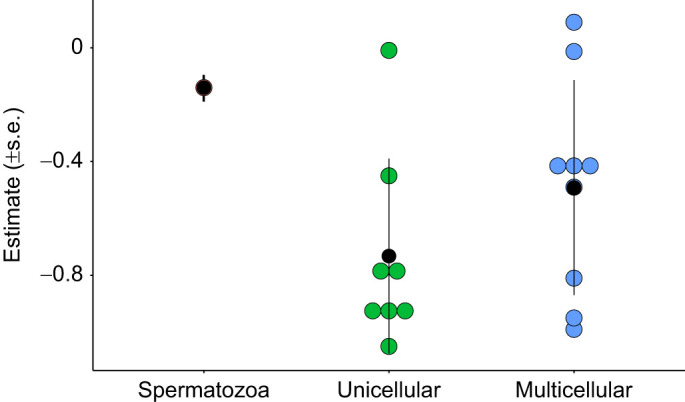
**Comparing mean scaling exponents for density-dependent metabolism.** Mean estimate of the scaling exponent for density-dependent metabolism for sperm compared with estimates from unicellular and multicellular species taken from several published sources (DeLong et al., 2014; Ghedini et al., 2017; Malerba et al., 2017; Marshall et al., 2022). The mean estimate of scaling of metabolism with density is shallower in sperm (*b*=−0.13) than most estimates for unicellular species (*b*=−1.05 to −0.009) and multicellular species (*b*=−3.05 to 0.09; note: one extreme scaling of −3.05 from the eusocial insect *Zacryptocerus pusillus* was removed from the plot for ease of view). Black dots are summary means (±s.e.m.) for each group.

Our comparative analysis combined with empirical observations suggest that density affects sperm metabolism across a wide range of taxa. We found a negative relationship between density and per capita metabolism – sperm at higher densities have lower per capita metabolic rates than sperm at lower densities. We show that the observed patterns are more likely caused by density-mediated changes to the sperm environment (e.g. pH, [CO_2_]) than resource limitation. Our results show that patterns of density-dependent metabolism extend beyond diploid metazoan phases into haploid phases (i.e. gametes) – both haploid and diploid phases can detect and modify their metabolism in response to their own density. However, when we compared our estimate of density-dependent metabolism with estimates found in whole organisms, we found that the relationship for sperm is shallower than most other estimates (DeLong et al., 2014; Ghedini et al., 2017; Malerba et al., 2017; Marshall et al., 2022) (Fig. 6). Our results indicate that sperm metabolism is highly plastic, similar to whole organisms, and shows greater variability than is appreciated.

Our results show a shallow, negative relationship between per capita metabolic rate and density (*b*=−0.13; Fig. 2A) both within and among species – sperm at lower densities have slightly higher individual metabolic rates than sperm at high densities. We find no support for resource limitation driving this pattern in that sperm diluted with carbohydrate-containing media had the same density-dependent metabolic response as sperm diluted with carbohydrate-free media (Fig. 4), similar to the hypothesis shown in Fig. 1C. Therefore, we interpret the results much the same way as the progenitors of the RDE, perceiving them as evidence for adaptive plasticity in sperm metabolism (Gray, 1928a; Rothschild, 1950, 1956). According to this hypothesis, sperm in dense suspensions have lower per capita metabolisms and longer lifespans than sperm in dilute suspensions (Chia and Bickell, 1983; Gray, 1928a; Levitan et al., 1991; Ohtake, 1976; Rothschild, 1956). Sperm remain energetically inactive within the dense matrix of the ejaculate, allowing them to conserve their limited energy reserves. Once diluted and free swimming, sperm increase their metabolism and expend energy to outcompete other sperm (i.e. within- or between-ejaculate competition) to fertilize eggs. For example, some internal fertilizers can store diluted sperm from multiple males at high densities in sperm storage organs before they fertilize eggs, increasing the risk of sperm competition (Simmons, 2002). In storage, sperm have decreased senescence, increased longevity and lower metabolic demands (Reinhardt, 2007; Ribou and Reinhardt, 2012). Once sperm reach the site of fertilization, they will rapidly increase their energy use, through greater motility and speed, to outcompete rival sperm for access to the ova (Simmons, 2002). Selection would not favour sperm that increased their metabolism while in storage, as this would increase senescence, decrease lifespan and sperm would likely expire before successfully reaching the ova. Similarly, having a reduced metabolism in a dilute suspension would mean that sperm may be too sluggish to compete for fertilizations. A large number of studies have demonstrated that sperm show adaptive changes in behaviour in response to local conditions (Alvarez et al., 2012; Babcock et al., 2014; Bahat and Eisenbach, 2006; Evans et al., 2012; Friedrich and Jülicher, 2007; Hadlow et al., 2023; Levitan et al., 1991); our results imply that this adaptive plasticity extends to metabolism.

Although our results indicate that density-dependent metabolism is not driven by resources, we still cannot rule out the possibility that resources play a role in the RDE. Increasing evidence suggests that female reproductive fluid (FRF) – i.e. fluid from the female reproductive tract or eggs – helps maintain sperm function (e.g. longevity, motility, physiology) across species (Gasparini et al., 2020). Seminal work from Gray (1928b) showed that metabolism and longevity increased in the presence of FRF (i.e. egg secretions) (Gray, 1928b). A more recent study by Hadlow et al. (2023) showed that age-related declines in sperm motility were somewhat alleviated by the addition of FRF (Hadlow et al., 2023). If FRF provides sperm with more per capita resources, this could explain higher per capita metabolisms at lower densities. An interesting addition to this research would be to investigate the unexplored effects of FRF on density-dependent sperm metabolism by diluting sperm with FRF and measuring metabolism at different concentrations in both internal and external fertilizers.

Our finding that the relationship between density and metabolism was the same regardless of diluent used (resource rich versus resource limited) implies that something other than resource availability is mediating metabolism. We, along with Gray, favour the hypothesis that the accumulation of metabolic products drives the relationship between sperm density and metabolism. Specifically, oxygen saturation, intracellular pH, carbon dioxide tension (Chia and Bickell, 1983), ion concentrations (e.g. bicarbonate and calcium; Pereira et al., 2017) and cell-to-cell communication (i.e. quorum sensing, Luther and Waberski, 2019; and sperm conjugation, Higginson and Pitnick, 2011; Monclus and Fornes, 2016) have all been suggested as cues, but some seem more likely than others. It is possible that oxygen limitation at high sperm concentrations depresses metabolic rate, but more than half of our estimates from published studies were at oxygen saturation levels that were greater than 50% [i.e. above most mammalian sperm critical *P*_O_2_ _levels (Marshall et al., 2013); see Table S4]. In our own empirical data, we do see evidence of a relationship between metabolic rate and oxygen saturation – metabolic rate (*V̇*_O_2__) decreases as oxygen saturation decreases for *G. caespitosa*. The rate of oxygen decline can also change based on: (1) the respirometer used – closed versus open; and (2) the sperm concentration measured – sperm at higher concentrations can deplete oxygen by 100% in a matter of minutes, whereas lower concentrations may take hours to deplete oxygen by just 20% (Table S4). Based on our data, we cannot rule out that sperm are oxy-conformers and reduce their oxygen uptake as the partial pressure of oxygen decreases (Marshall et al., 2013; Prosser, 1973), but this requires further explicit study.

We suspect that density-mediated changes in the pH and CO_2_ concentration of the sperm environment drive the changes in metabolism that we observed, given the role of both in mediating sperm activity and function more generally. In high-density conditions, sperm tend to generate more CO_2_ per volume, resulting in lower HCO_3_^−^/pH_i_ and decreased activity. However, as sperm dilute into environments with elevated HCO_3_^−^/pH_i_, the spatial dispersion leads to reduced CO_2_ levels per volume. As sperm transition from a state of low activity (higher densities) in the male reproductive organ to a state of higher activity (lower densities) in the female tract or aquatic environment, changes in the external environment, such as elevated HCO_3_^−^ levels and pH, contribute to the initiation of sperm capacitation (Tresguerres et al., 2010). Sperm capacitation is a complex process that is crucial for fertilization and is initiated by various factors including CO_2_, bicarbonate ions (HCO_3_^−^), intracellular pH (pH_i_) regulation and ATP dynamics (Chia and Bickell, 1983; Cohn, 1918; Foley and Williams, 1967; Grahn et al., 2023; Mohri and Yasumasu, 1963; Rothschild, 1956; Speer et al., 2021; Tresguerres et al., 2010; Zhou et al., 2015). HCO_3_^−^:CO_2_ plays a pivotal role in capacitation through intracellular alkalinization, resulting in an elevated pH_i_, triggering hyperactivation (i.e. increase flagellar beating) (Grahn et al., 2023). This process is likely conserved across mammals and free-spawning marine invertebrates, influencing flagellar beating and fertilization (Beltrán et al., 2007; Boulais et al., 2019; Christen et al., 1983; Grahn et al., 2023; Hess et al., 2005; Qi et al., 2007; Rothschild, 1956; Speer et al., 2021; Vacquier et al., 2014; Wang et al., 2007). Additionally, elevated intracellular HCO_3_^−^, similar to levels found in reproductive fluids, activates soluble adenylyl cyclase (sAC), which uses ATP to trigger ﻿cyclic adenosine 3′,5'-monophosphate (cAMP) signalling pathways (Chen et al., 2000; Hess et al., 2005; Wandernoth et al., 2010), stimulating sperm motility (Wang et al., 2007) and respiration (Foley and Williams, 1967; Tajima et al., 1987). An important next step would be to disentangle CO_2_ and pH from sperm density to determine exactly which covarying component drives sperm metabolism.

We found that sperm from endotherms have higher metabolism than sperm from ectotherms – metabolic rates are ∼2-fold greater in endotherms. This is similar to patterns seen in whole organisms, though the effect was less strong: mass-specific metabolic rates can be ∼6-fold higher for endotherms (Gillooly et al., 2017). Endotherms exhibit larger body sizes, higher internal temperatures and internal fertilization. Internal fertilizers produce much larger sperm (Kahrl et al., 2021) with a longer flagellum (i.e. tail+midpiece), and evidence suggests that larger sperm can swim faster (Fitzpatrick et al., 2009; Gomendio and Roldan, 1991, 2008; Simpson et al., 2014), implying a potential increase in sperm energy expenditure. Given that endotherms have sperm with higher metabolisms, and therefore higher rates of CO_2_ production, they should show a steeper density–metabolism relationship relative to ectotherms. Instead, we found that endothermic sperm show similar density–metabolism relationships as ectotherms, implying that the way in which environmental changes alter density-dependent metabolism in sperm may be conserved across groups. Although we still favour the role of CO_2_/HCO_3_^−^/pH_i_ as an explanation, another plausible explanation for density–metabolism relationships in sperm is that sperm are activated by mechanical dilution whereby sperm become surrounded by free space once they are diluted, which provides room for movement and allows them to expend more energy than those in dense suspensions – termed allelostasis (Gray, 1928a).

Evidence is mixed regarding the relationship between sperm metabolism and sperm activity (i.e. motility and velocity) across species. Some studies have found a link between metabolism and sperm activity (Boell and Burkus, 1984; Burness et al., 2004, 2005; Lahnsteiner et al., 1996, 1999) whereas others have found no relationship (Bernardini et al., 1988; Burness et al., 2005; Cardullo and Cone, 1986; Deutch et al., 1985; Lahnsteiner and Caberlotto, 2012; Murdoch et al., 1999). Additionally, swimming speed positively covaries with sperm density in some species (Reinhardt and Otti, 2012) but not others (Ginsburg and Armant, 1990; Levitan, 2000). Based on these studies and the negative density-dependent pattern we observed in our data, two things may be possible: either (1) the relationship is driven by reduced motility, where a significant proportion of sperm exhibit minimal movement at higher densities, resulting in lower oxygen consumption rates, or (2) it is driven by reduced velocity, where numerous sperm display sluggish movement, leading to lower oxygen consumption rates. We examined a subset of studies in our compilation and a few external studies to examine the relationship between percent motility and concentration across species. We found a strong positive relationship (*F*_1,69_=5.47, *P*=0.02, coefficient±s.e.=3.10±1.30) – a higher percentage of sperm are moving in higher concentrations – the opposite of what would be expected if motility was driving the sperm concentration–metabolism relationship. Therefore, based on the limited data available, it seems more likely that sperm velocity negatively covaries with concentration such that a higher percentage of sperm are moving at higher densities, but they are moving less quickly than in lower densities. We do not have enough data to test this inference as things stand, but believe it is an important next step for identifying the proximal drivers of the sperm metabolic plasticity.

### Consequences of our findings

A practical consequence of our discovery that the RDE is widespread across the tree of life is that many of the standardized sperm metabolic rates reported in the literature are incorrect. A tradition in the field of sperm metabolic research is to measure sperm metabolism at whatever concentration is practical and then use a linear multiplier to convert that metabolic rate to the predicted metabolic rate (i.e. *Z*_O_2__) at a common concentration based on a standardized sperm concentration (Redenz, 1933). For example, studies of mammals typically convert their estimates of metabolism to those expected at a concentration of 10^8^ sperm ml^−1^ (see Table S1)*.* Garrett et al. (2008) measured metabolism of freshly collected bull (*Bos taurus*) sperm at a concentration of 5×10^7^ sperm ml^−1^, but expressed metabolism in 10^8^ sperm ml^−1^. A nonlinear relationship between sperm metabolism and concentration means that using a linear conversion introduces systematic error. Simply put, when the test concentration is higher than the standardized reported concentration, such conversions underestimate the true metabolic rate; when the test concentration is lower than the standardized reported concentration, such conversions overestimate metabolic rate. As Fig. 5 shows, these misestimates can be substantial – across all the studies considered, metabolic rates can be misestimated by up to 5-fold. We suggest that future studies report the sperm metabolic rate at the concentration at which it was measured, and report the metabolic rate at that concentration without conversion to a standardized metabolic rate.

Although our analyses reveal general and consistent patterns of density-dependent sperm metabolism, some key uncertainties remain. First, sperm have finite energy reserves such that there should be a trade-off between sperm function (i.e. metabolic rate and velocity) and duration of energy use (longevity) – any increase in one will likely cause a decrease in the other (Ball and Parker, 1996; Pizzari and Parker, 2009). Sperm size has also been linked to swimming speed (and thus energy use), but the direction and magnitude of this relationship is variable (positive, negative or no relationship) and species-specific (Gomendio and Roldan, 1991, 2008; Lamunyon and Ward, 1998; Malo et al., 2006; Simpson et al., 2014). Determining how sperm size, swimming speed and longevity all interact with sperm concentration to affect metabolism is an important next step. Second, some internally fertilizing species can store diluted sperm in the female reproductive tract for extended periods of time (days to years) (Holt and Fazeli, 2016). Sperm that are densely packed into confined spaces within the female reproductive tract may be shielded from the effects of dilution (i.e. respiration and ageing) until they reach the site of fertilization (Reinhardt, 2007). A critical next step would be to explore how the RDE differs between internal fertilizers that store sperm versus those that do not. Finally, it would be interesting to determine whether there is similar density-dependent metabolism in externally shed female gametes (i.e. eggs). If eggs are less energy limited, which seems likely, we might expect that metabolism is unaffected by density. In contrast, the relatively small surface area to volume ratio of eggs means that oxygen availability can reduce egg survival (Hendry and Day, 2003; Seymour and White, 2006). Thus, examining egg density–metabolism relationships seems like an interesting topic for further exploration.

## Supplementary Material

10.1242/jexbio.246674_sup1 Supplementary information

## References

[JEB246674C1] Akaike, H. (1973). Maximum likelihood identification of GSussian autoregressive moving average models. *Biometrika* 60, 255-265. 10.1093/biomet/60.2.255

[JEB246674C2] Alvarez, L., Dai, L., Friedrich, B. M., Kashikar, N. D., Gregor, I., Pascal, R. and Kaupp, U. B. (2012). The rate of change in Ca^2+^ concentration controls sperm chemotaxis. *J. Cell Biol.* 196, 653-663. 10.1083/jcb.201106096 PMC3307702 22371558

[JEB246674C3] Amundsen, P. A., Knudsen, R. and Klemetsen, A. (2007). Intraspecific competition and density dependence of food consumption and growth in Arctic charr. *J. Anim. Ecol.* 76, 149-158. 10.1111/j.1365-2656.2006.01179.x 17184363

[JEB246674C4] Auer, S. K., Salin, K., Rudolf, A. M., Anderson, G. J. and Metcalfe, N. B. (2015). Flexibility in metabolic rate confers a growth advantage under changing food availability. *J. Anim. Ecol.* 84, 1405-1411. 10.1111/1365-2656.12384 PMC4682473 25939669

[JEB246674C5] Babcock, D. F., Wandernoth, P. M. and Wennemuth, G. (2014). Episodic rolling and transient attachments create diversity in sperm swimming behavior. *BMC Biol.* 12, 67. 10.1186/s12915-014-0067-3 25182562 PMC4354980

[JEB246674C6] Bahat, A. and Eisenbach, M. (2006). Sperm thermotaxis. *Mol. Cell. Endocrinol.* 252, 115-119. 10.1016/j.mce.2006.03.027 16672171

[JEB246674C7] Ball, M. A. and Parker, G. A. (1996). Sperm competition games: external fertilization and ‘adaptive’ infertility. *J. Theor. Biol.* 180, 141-150. 10.1006/jtbi.1996.0090 8763365

[JEB246674C8] Barneche, D. R., Robertson, D. R., White, C. R. and Marshall, D. J. (2018). Fish reproductive-energy output increases disproportionately with body size. *Science* 360, 642-645. 10.1126/science.aao6868 29748282

[JEB246674C9] Beltrán, C., Vacquier, V. D., Moy, G., Chen, Y., Buck, J., Levin, L. R. and Darszon, A. (2007). Particulate and soluble adenylyl cyclases participate in the sperm acrosome reaction. *Biochem. Biophys. Res. Commun.* 358, 1128-1135. 10.1016/j.bbrc.2007.05.061 17524362 PMC3644950

[JEB246674C10] Bernardini, G., Belgiojoso, P. and Camatini, M. (1988). Xenopus spermatozoon: is there any correlation between motility and oxygen consumption? *Gamete Res.* 21, 403-408. 10.1002/mrd.1120210408 2464532

[JEB246674C11] Boell, E. J. and Burkus, J. K. (1984). Oxygen consumption and motility of mouse sperm as affected by oxidizable substrates and oxygen tension. *Carlsberg Res. Commun.* 49, 147-154. 10.1007/BF02913942

[JEB246674C12] Borenstein, M., Hedges, L. V., Higgins, J. P. T. and Rothstein, H. (2009). *Introduction to Meta-Analysis*. John Wiley & Sons.

[JEB246674C13] Boulais, M., Demoy-Schneider, M., Alavi, S. M. H. and Cosson, J. (2019). Spermatozoa motility in bivalves: Signaling, flagellar beating behavior, and energetics. *Theriogenology* 136, 15-27. 10.1016/j.theriogenology.2019.06.025 31234053

[JEB246674C14] Burness, G., Casselman, S. J., Schulte-Hostedde, A. I., Moyes, C. D. and Montgomerie, R. (2004). Sperm swimming speed and energetics vary with sperm competition risk in bluegill (*Lepomis macrochirus*). *Behav. Ecol. Sociobiol.* 56, 65-70. 10.1007/s00265-003-0752-7

[JEB246674C15] Burness, G., Moyes, C. D. and Montgomerie, R. (2005). Motility, ATP levels and metabolic enzyme activity of sperm from bluegill (*Lepomis macrochirus*). *Comp. Biochem. Physiol.* 140, 11-17. 10.1016/j.cbpb.2004.09.021 15664308

[JEB246674C16] Cameron, J. N. (1986). Solubility of O_2_ and CO_2_ at different temperatures and salinities (appendix table). In *Principles of Physiological Measurement*, pp. 254-259. Academic Press.

[JEB246674C17] Cardullo, R. A. and Cone, R. A. (1986). Mechanical immobilization of rat sperm does not change their oxygen consumption rate. *Biol. Reprod.* 34, 820-830. 10.1095/biolreprod34.5.820 3730479

[JEB246674C18] Chen, Y., Cann, M. J., Litvin, T. N., Iourgenko, V., Sinclair, M. L., Levin, L. R. and Buck, J. (2000). Soluble adenylyl cyclase as an evolutionarily conserved bicarbonate sensor. *Science (80-.)* 289, 625-628. 10.1126/science.289.5479.625 10915626

[JEB246674C19] Chia, F. S. and Bickell, L. R. (1983). Echinodermata. In *Reproductive Biology of Invertebrates. V. 2. Spermatogenesis and Sperm Function* (ed. K. G. Adiyodi and R. G. Adiyodi.), pp. 545-620. Wiley.

[JEB246674C20] Christen, R., Schackmann, R. W. and Shapiro, B. M. (1983). Metabolism of sea urchin sperm. Interrelationships between intracellular pH, ATPase activity, and mitochondrial respiration. *J. Biol. Chem.* 258, 5392-5399. 10.1016/S0021-9258(20)81902-4 6222053

[JEB246674C21] Cohn, E. J. (1918). Studies in the physiology of spermatozoa. *Biol. Bull.* 34, 167-218. 10.2307/1536264

[JEB246674C22] Cornman, I. (1941). Sperm activation by *Arbacia* egg extracts, with special reference to echinochrome. *Biol. Bull.* 80, 202-207. 10.2307/1537598

[JEB246674C23] DeLong, J. P. and Hanson, D. T. (2009). Density-dependent individual and population-level metabolic rates in a suite of single-celled eukaryotes. *Open Biol. J.* 2, 32-37. 10.2174/1874196700902010032

[JEB246674C24] DeLong, J. P., Hanley, T. C. and Vasseur, D. A. (2014). Competition and the density dependence of metabolic rates. *J. Anim. Ecol.* 83, 51-58. 10.1111/1365-2656.12065 23565624

[JEB246674C25] Deutch, D. S., Katz, D. F. and Overstreet, J. W. (1985). Increases in human sperm oxygen consumption at low cell concentrations. *Biol. Reprod.* 32, 865-871. 10.1095/biolreprod32.4.865 4005350

[JEB246674C26] Dreanno, C., Cosson, J., Suquet, M., Cibert, C., Fauvel, C., Dorange, G. and Billard, R. (1999). Effects of osmolality, morphology perturbations and intracellular nucleotide content during the movement of sea bass (*Dicentrarchus labrax*) spermatozoa. *J. Reprod. Fertil.* 116, 113-125. 10.1530/jrf.0.1160113 10505062

[JEB246674C27] Evans, J. P., Garcia-Gonzalez, F., Almbro, M., Robinson, O. and Fitzpatrick, J. L. (2012). Assessing the potential for egg chemoattractants to mediate sexual selection in a broadcast spawning marine invertebrate. *Proc. R. Soc. B Biol. Sci.* 279, 2855-2861. 10.1098/rspb.2012.0181 PMC3367782 22438495

[JEB246674C28] Fitzpatrick, J. L., Montgomerie, R., Desjardins, J., Stiver, K. A., Kolm, N. and Balshine, S. (2009). Female promiscuity promotes the evolution of faster sperm in cichlid fishes. *Proc. Natl. Acad. Sci. USA* 106, 1128-1132. 10.1073/pnas.0809990106 PMC2633556 19164576

[JEB246674C29] Foley, C. W. and Williams, W. L. (1967). Effect of bicarbonate and oviduct fluid on respiration of spermatozoa. *Proc. Soc. Exp. Biol. Med.* 126, 634-637. 10.3181/00379727-126-32526

[JEB246674C30] Friedrich, B. M. and Jülicher, F. (2007). Chemotaxis of sperm cells. *Proc. Natl. Acad. Sci. USA* 104, 13256-13261. 10.1073/pnas.0703530104 PMC1948934 17686980

[JEB246674C31] Garrett, L. J. A., Revell, S. G. and Leese, H. J. (2008). Adenosine triphosphate production by bovine spermatozoa and its relationship to semen fertilizing ability. *J. Androl.* 29, 449-458. 10.2164/jandrol.107.003533 18046050

[JEB246674C32] Gasparini, C., Pilastro, A. and Evans, J. P. (2020). The role of female reproductive fluid in sperm competition: FRF and fertilization bias. *Philos. Trans. R. Soc. B Biol. Sci.* 375, 20200077. 10.1098/rstb.2020.0077 PMC7661459 33070736

[JEB246674C33] Ghedini, G., White, C. R. and Marshall, D. J. (2017). Does energy flux predict density-dependence? An empirical field test. *Ecology* 98, 3116-3126. 10.1002/ecy.2033 28950411

[JEB246674C34] Gillooly, J. F., Brown, J. H., West, G. B., Savage, V. M. and Charnov, E. L. (2001). Effects of size and temperature on metabolic rate. *Science* 293, 2248-2251. 10.1126/science.1061967 11567137

[JEB246674C35] Gillooly, J. F., Gomez, J. P. and Mavrodiev, E. V. (2017). A broad-scale comparison of aerobic activity levels in vertebrates: endotherms versus ectotherms. *Proc. R. Soc. B Biol. Sci.* 284, 20162328. 10.1098/rspb.2016.2328 PMC5326522 28202808

[JEB246674C36] Ginsburg, K. A. and Armant, R. D. (1990). The influence of chamber characteristics on the reliability of sperm concentration and movement measurements obtained by manual and videomicrographic analysis. *Fertil. Steril.* 53, 882-887. 10.1016/S0015-0282(16)53526-2 2332061

[JEB246674C37] Glazier, D. S. (2008). Effects of metabolic level on the body size scaling of metabolic rate in birds and mammals. *Proc. R. Soc. B Biol. Sci.* 275, 1405-1410. 10.1098/rspb.2008.0118 PMC2602708 18348961

[JEB246674C38] Glazier, D. S. (2010). A unifying explanation for diverse metabolic scaling in animals and plants. *Biol. Rev.* 85, 111-138. 10.1111/j.1469-185X.2009.00095.x 19895606

[JEB246674C39] Gomendio, M. and Roldan, E. R. S. (1991). Sperm competition influences sperm size in mammals. *Proc. R. Soc. B Biol. Sci.* 243, 181-185. 10.1098/rspb.1991.0029 1675796

[JEB246674C40] Gomendio, M. and Roldan, E. R. S. (2008). Implications of diversity in sperm size and function for sperm competition and fertility. *Int. J. Dev. Biol.* 52, 439-447. 10.1387/ijdb.082595mg 18649256

[JEB246674C41] Grahn, E., Kaufmann, S. V., Askarova, M., Ninov, M., Welp, L. M., Berger, T. K., Urlaub, H. and Kaupp, U. B. (2023). Control of intracellular pH and bicarbonate by CO_2_ diffusion into human sperm. *Nat. Commun.* 14, 5395. 10.1038/s41467-023-40855-0 37669933 PMC10480191

[JEB246674C42] Gray, J. (1928a). The effect of dilution on the activity of spermatozoa. *J. Exp. Biol.* 5, 337-344. 10.1242/jeb.5.4.337

[JEB246674C43] Gray, J. (1928b). The effect of egg-secretions on the activity of spermatozoa. *J. Exp. Biol.* 5, 362-365. 10.1242/jeb.5.4.362

[JEB246674C44] Gray, J. (1928c). The senescence of spermatozoa. *J. Exp. Biol.* 5, 345-361. 10.1242/jeb.5.4.345

[JEB246674C45] Hadlow, J. H., Evans, J. P. and Lymbery, R. A. (2023). Female reproductive fluids ‘rescue’ sperm from phenotypic ageing in an external fertilizer. *Proc. R. Soc. B Biol. Sci.* 290, 20230574. 10.1098/rspb.2023.0574 PMC10206448 37221848

[JEB246674C46] Hathaway, R. R. (1963). Activation of respiration in sea urchin spermatozoa by egg water. *Biol. Bull.* 125, 486-498. 10.2307/1539361

[JEB246674C47] Hendry, A. P. and Day, T. (2003). Revisiting the positive correlation between female size and egg size. *Evol. Ecol. Res.* 5, 421-429.

[JEB246674C48] Hess, K. C., Jones, B. H., Marquez, B., Chen, Y., Ord, T. S., Kamenetsky, M., Miyamoto, C., Zippin, J. H., Kopf, G. S., Suarez, S. S. et al. (2005). The ‘soluble’ adenylyl cyclase in sperm mediates multiple signaling events required for fertilization. *Dev. Cell* 9, 249-259. 10.1016/j.devcel.2005.06.007 16054031 PMC3082461

[JEB246674C49] Higginson, D. M. and Pitnick, S. (2011). Evolution of intra-ejaculate sperm interactions: do sperm cooperate? *Biol. Rev.* 86, 249-270. 10.1111/j.1469-185X.2010.00147.x 20608927

[JEB246674C50] Holt, W. V. and Fazeli, A. (2016). Sperm storage in the female reproductive tract. *Annu. Rev. Anim. Biosci.* 4, 291-310. 10.1146/annurev-animal-021815-111350 26526545

[JEB246674C51] Ives, A. R. (2018). *Mixed and Phylogenetic Models: A Conceptual Introduction to Correlated Data*. Victoria, Canada: Leanpub.

[JEB246674C52] Jones, R. C. and Murdoch, R. N. (1996). Regulation of the motility and metabolism of spermatozoa for storage in the epididymis of eutherian and marsupial mammals. *Reprod. Fertil. Dev.* 8, 553-568. 10.1071/RD9960553 8870080

[JEB246674C53] Kahrl, A. F., Snook, R. R. and Fitzpatrick, J. L. (2021). Fertilization mode drives sperm length evolution across the animal tree of life. *Nat. Ecol. Evol.* 5, 1153-1164. 10.1038/s41559-021-01488-y 34155385

[JEB246674C54] Lahnsteiner, F. and Caberlotto, S. (2012). Motility of gilthead seabream *Sparus aurata* spermatozoa and its relation to temperature, energy metabolism and oxidative stress. *Aquaculture* 370-371, 76-83. 10.1016/j.aquaculture.2012.09.034

[JEB246674C55] Lahnsteiner, F., Berger, B., Weismann, T. and Patzner, R. A. (1996). Motility of spermatozoa of *Alburnus alburnus* (Cyprinidae) and its relationship to seminal plasma composition and sperm metabolism. *Fish Physiol. Biochem.* 15, 167-179. 10.1007/BF01875596 24194090

[JEB246674C56] Lahnsteiner, F., Berger, B. and Weismann, T. (1999). Sperm metabolism of the teleost fishes *Chalcalburnus chalcoides* and *Oncorhynchus mykiss* and its relation to motility and viability. *J. Exp. Zool.* 284, 454-465. 10.1002/(SICI)1097-010X(19990901)284:4<454::AID-JEZ12>3.0.CO;2-O 10451423

[JEB246674C57] Lamunyon, C. W. and Ward, S. (1998). Larger sperm outcompete smaller sperm in the nematode *Caenorhabditis elegans*. *Proc R Soc* 265, 1997-2002. 10.1098/rspb.1998.0531 PMC1689481 9821364

[JEB246674C58] Levitan, D. R. (2000). Sperm velocity and longevity trade off each other and influence fertilization in the sea urchin *Lytechinus variegatus*. *Proc. R. Soc. B Biol. Sci.* 267, 531-534. 10.1098/rspb.2000.1032 PMC1690568 10787153

[JEB246674C59] Levitan, D. R., Sewell, M. A. and Chia, F.-S. (1991). Kinetics of fertilization in the sea urchin *Strongylocentrotus franciscanus*: interaction of gamete dilution, age, and contact time. *Biol. Bull.* 181, 371-378. 10.2307/1542357 29304673

[JEB246674C60] Lillie, F. R. (1913). Studies of fertilization. V. The behavior of the spermatozoa of *Nereis* and *Arbacia* with special reference to egg-extractives. *J. Exp. Zool.* 14, 515-574. 10.1002/jez.1400140403

[JEB246674C61] Lovass, M. K., Marshall, D. J. and Ghedini, G. (2020). Conspecific chemical cues drive density-dependent metabolic suppression independently of resource intake. *J. Exp. Biol.* 223, jeb224824. 10.1242/jeb.224824 32709627

[JEB246674C62] Luther, A. M. and Waberski, D. (2019). *In vitro* aging of boar spermatozoa: role of sperm proximity and seminal plasma. *Andrology* 7, 382-390. 10.1111/andr.12600 30793513

[JEB246674C63] Malerba, M. E., White, C. R. and Marshall, D. J. (2017). Phytoplankton size-scaling of net-energy flux across light and biomass gradients. *Ecology* 98, 3106-3115. 10.1002/ecy.2032 28940445

[JEB246674C64] Malo, A. F., Gomendio, M., Garde, J., Lang-Lenton, B., Soler, A. J. and Roldan, E. R. S. (2006). Sperm design and sperm function. *Biol. Lett.* 2, 246-249. 10.1098/rsbl.2006.0449 17148374 PMC1618917

[JEB246674C65] Mansour, N., Lahnsteiner, F. and Berger, B. (2003). Metabolism of intratesticular spermatozoa of a tropical teleost fish (*Clarias gariepinus*). *Comp. Biochem. Physiol. B* 135, 285-296. 10.1016/S1096-4959(03)00083-6 12798939

[JEB246674C66] Marshall, D. J. and Evans, J. P. (2005). Does egg competition occur in marine broadcast spawners? *J. Evol. Biol.* 18, 1244-1252. 10.1111/j.1420-9101.2005.00947.x 16135120

[JEB246674C67] Marshall, D. J., Bode, M. and White, C. R. (2013). Estimating physiological tolerances: a comparison of traditional approaches to nonlinear regression techniques. *J. Exp. Biol.* 216, 2176-2182. 10.1242/jeb.085712 23470657

[JEB246674C68] Marshall, D. J., Pettersen, A. K., Bode, M. and White, C. R. (2020). Developmental cost theory predicts thermal environment and vulnerability to global warming. *Nat. Ecol. Evol.* 4, 406-411. 10.1038/s41559-020-1114-9 32127682

[JEB246674C69] Marshall, D. J., Malerba, M., Lines, T., Sezmis, A. L., Hasan, C. M., Lenski, R. E. and Mcdonald, M. J. (2022). Long-term experimental evolution decouples size and production costs in *Escherichia coli*. *Proc. Natl. Acad. Sci. USA* 119, 1-8. 10.1073/pnas.2200713119 PMC9173777 35594402

[JEB246674C70] Massino, C., Wetzker, C., Balvin, O., Bartonicka, T., Kremenova, J., Sasinkova, M., Otti, O. and Reinhardt, K. (2021). Seminal fluid and sperm diluent affect sperm metabolism in an insect: evidence from NAD(P)H and flavin adenine dinucleotide autofluorescence lifetime imaging. *Microsc. Res. Tech.* 85, 398-411. 10.1002/jemt.23914 34486193

[JEB246674C71] Michonneau, F., Brown, J. W. and Winter, D. J. (2016). rotl: an R package to interact with the Open Tree of Life data. *Methods Ecol. Evol.* 7, 1476-1481. 10.1111/2041-210X.12593

[JEB246674C72] Moher, D., Shamseer, L., Clarke, M., Ghersi, D., Liberati, A., Petticrew, M., Shekelle, P., Stewart, L. A., Estarli, M., Barrera, E. S. A. et al. (2016). Preferred reporting items for systematic review and meta-analysis protocols (PRISMA-P) 2015 statement. *Rev. Esp. Nutr. Humana y Diet.* 20, 148-160. 10.14306/renhyd.20.2.223

[JEB246674C73] Mohri, H. and Yasumasu, I. (1963). Studies on the respiration of sea-urchin spermatozoa V. The effect of *P*_CO_2__. *J. Exp. Biol.* 40, 573-586. 10.1242/jeb.40.4.573 14086808

[JEB246674C74] Monclus, M. A. and Fornes, M. W. (2016). Sperm conjugation in mammal reproductive function: different names for the same phenomenon? *Mol. Reprod. Dev.* 83, 884-896. 10.1002/mrd.22636 26970336

[JEB246674C75] Murdoch, R. N., Armstrong, V. L., Clulow, J. and Jones, R. C. (1999). Relationship between motility and oxygen consumption of sperm from the cauda epididymides of the rat. *Reprod. Fertil. Dev.* 11, 87-94. 10.1071/RD99039 10735552

[JEB246674C76] O′Dea, R. E., Lagisz, M., Jennions, M. D., Koricheva, J., Noble, D. W. A., Parker, T. H., Gurevitch, J., Page, M. J., Stewart, G., Moher, D. et al. (2021). Preferred reporting items for systematic reviews and meta-analyses in ecology and evolutionary biology: a PRISMA extension. *Biol. Rev.* 96, 1695-1722. 10.1111/brv.12721 PMC8518748 33960637

[JEB246674C77] Ohtake, H. (1976). Respiratory behaviour of sea-urchin spermatozoa. I. Effect of pH and egg water on the respiratory rate. *J. Exp. Zool.* 198, 303-311. 10.1002/jez.1401980303 12246

[JEB246674C78] Olito, C., White, C. R., Marshall, D. J. and Barneche, D. R. (2017). Estimating monotonic rates from biological data using local linear regression. *J. Exp. Biol.* 220, 759-764. 10.1242/jeb.148775 28049626

[JEB246674C109] Ouzzani, M., Hammady, H., Fedorowicz, Z. and Elmagarmid, A. (2016). Rayyan-a web and mobile app for systematic reviews. *Systematic Reviews* 5, 1-10. 10.1186/s13643-016-0384-4 PMC5139140 27919275

[JEB246674C79] Parker, G. A. (1993). Sperm competition games: sperm size and sperm number under adult control. *Proc. R. Soc. B Biol. Sci.* 253, 245-254. 10.1098/rspb.1993.0110 8234363

[JEB246674C80] Pereira, R., Sá, R., Barros, A. and Sousa, M. (2017). Major regulatory mechanisms involved in sperm motility. *Asian J. Androl.* 19, 5-14. 10.4103/1008-682X.167716 PMC5227674 26680031

[JEB246674C81] Pettersen, A. K., White, C. R., Bryson-Richardson, R. J. and Marshall, D. J. (2019). Linking life-history theory and metabolic theory explains the offspring size–temperature relationship. *Ecol. Lett.* 22, 518-526. 10.1111/ele.13213 30618178

[JEB246674C82] Pizzari, T. and Parker, G. A. (2009). Sperm competition and sperm phenotype. In *Sperm Biology* (ed. T. R. Birkhead, D. J. Hosken, S. Pitnick), pp. 207-245. Elsevier Ltd.

[JEB246674C83] Poli, F., Immler, S. and Gasparini, C. (2019). Effects of ovarian fluid on sperm traits and its implications for cryptic female choice in zebrafish. *Behav. Ecol.* 30, 1298-1305. 10.1093/beheco/arz077

[JEB246674C84] Potter, A., White, C., and Marshall, D. (2024). Per capita sperm metabolism is density dependent. *Dataset. Dryad.* 10.5061/dryad.hhmgqnkm1 PMC11006396 38380562

[JEB246674C85] Prosser, C. L. (1973). *Comparative Animal Physiology*. W.B. Saunders Company.

[JEB246674C86] Qi, H., Moran, M. M., Navarro, B., Chong, J. A., Krapivinsky, G., Krapivinsky, L., Kirichok, Y., Ramsey, I. S., Quill, T. A. and Clapham, D. E. (2007). All four CatSper ion channel proteins are required for male fertility and sperm cell hyperactivated motility. *Proc. Natl. Acad. Sci. USA* 104, 1219-1223. 10.1073/pnas.0610286104 PMC1770895 17227845

[JEB246674C87] Rahi, D., Dzyuba, B., Xin, M., Cheng, Y. and Dzyuba, V. (2020). Energy pathways associated with sustained spermatozoon motility in the endangered Siberian sturgeon *Acipenser baerii*. *J. Fish Biol.* 97, 435-443. 10.1111/jfb.14382 32415790

[JEB246674C88] Redenz, E. (1933). Über den spaltungsstoffwechsel der säugetierspermatozoen im zusammenhang mit der beweglichkeit. *Biochem. Z.* 257, 234-241.

[JEB246674C89] Reinhardt, K. (2007). Evolutionary consequences of sperm cell aging. *Q. Rev. Biol.* 82, 375-393. 10.1086/522811 18217528

[JEB246674C90] Reinhardt, K. and Otti, O. (2012). Comparing sperm swimming speed. *Evol. Ecol. Res.* 14, 1-18.

[JEB246674C91] Ribou, A. C. and Reinhardt, K. (2012). Reduced metabolic rate and oxygen radicals production in stored insect sperm. *Proc. R. Soc. B Biol. Sci.* 279, 2196-2203. 10.1098/rspb.2011.2422 PMC3321705 22279170

[JEB246674C92] Rohatgi, A. (2020). *WebPlotDigitizer User Manual Version 4.3*. https://automeris.io/WebPlotDigitizer

[JEB246674C93] Rothschild, L. (1948). The physiology of sea-urchin spermatozoa: senescence and the dilution effect. *J. Exp. Biol.* 25, 353-368. 10.1242/jeb.25.4.353 18115871

[JEB246674C94] Rothschild, L. (1950). The respiration of sea-urchin spermatozoa. *J. Exp. Biol.* 27, 420-436. 10.1242/jeb.27.3.420 14803623

[JEB246674C95] Rothschild, L. (1956). The respiratory dilution effect in sea-urchin spermatozoa. *Vie Milieu* 7, 405-412.

[JEB246674C96] Ruiz-Pesini, E., Díez-Sánchez, C., López-Pérez, M. J. and Enríquez, J. A. (2007). The role of the mitochondrion in sperm function: is there a place for oxidative phosphorylation or is this a purely glycolytic process? *Curr. Top. Dev. Biol.* 77, 3-19. 10.1016/S0070-2153(06)77001-6 17222698

[JEB246674C97] Seymour, R. S. and White, C. R. (2006). Models for embryonic respiration. In *Comparative Developmental Physiology: Contributions, Tools, and Trends*, pp. 41-57. Oxford University Press.

[JEB246674C98] Simmons, L. W. (2002). *Sperm Competition and Its Evolutionary Consequences in the Insects*. Princeton University Press.

[JEB246674C99] Simpson, J. L., Humphries, S., Evans, J. P., Simmons, L. W. and Fitzpatrick, J. L. (2014). Relationships between sperm length and speed differ among three internally and three externally fertilizing species. *Evolution (N. Y)* 68, 92-104. 10.1111/evo.12199 24224469

[JEB246674C100] Speer, K. F., Allen-Waller, L., Novikov, D. R. and Barott, K. L. (2021). Molecular mechanisms of sperm motility are conserved in a basal metazoan. *Proc. Natl. Acad. Sci. USA* 118, e2109993118. 10.1073/pnas.2109993118 34810263 PMC8640785

[JEB246674C101] Tajima, Y., Okamura, N. and Sugita, Y. (1987). The activating effects of bicarbonate on sperm motility and respiration at ejaculation. *BBA Gen. Subj.* 924, 519-529. 10.1016/0304-4165(87)90168-1 3036242

[JEB246674C102] Tresguerres, M., Buck, J. and Levin, L. R. (2010). Physiological carbon dioxide, bicarbonate, and pH sensing. *Pflugers Arch. Eur. J. Physiol.* 460, 953-964. 10.1007/s00424-010-0865-6 20683624 PMC2967379

[JEB246674C103] Vacquier, V. D., Loza-Huerta, A., García-Rincón, J., Darszon, A. and Beltrán, C. (2014). Soluble adenylyl cyclase of sea urchin spermatozoa. *Biochim. Biophys. Acta Mol. Basis Dis.* 1842, 2621-2628. 10.1016/j.bbadis.2014.07.011 PMC4262560 25064590

[JEB246674C104] Wandernoth, P. M., Raubuch, M., Mannowetz, N., Becker, H. M., Deitmer, J. W., Sly, W. S. and Wennemuth, G. (2010). Role of carbonic anhydrase IV in the bicarbonate-mediated activation of murine and human sperm. *PLoS One* 5, e15061. 10.1371/journal.pone.0015061 PMC2991337 21124840

[JEB246674C105] Wang, D., Hu, J., Bobulescu, I. A., Quill, T. A., McLeroy, P., Moe, O. W. and Garbers, D. L. (2007). A sperm-specific Na^+^/H^+^ exchanger (sNHE) is critical for expression and in vivo bicarbonate regulation of the soluble adenylyl cyclase (sAC). *Proc. Natl. Acad. Sci. USA* 104, 9325-9330. 10.1073/pnas.0611296104 PMC1890493 17517652

[JEB246674C106] White, C. R., Kearney, M. R., Matthews, P. G. D., Kooijman, S. A. L. M. and Marshall, D. J. (2011). A manipulative test of competing theories for metabolic scaling. *Am. Nat.* 178, 746-754. 10.1086/662666 22089869

[JEB246674C107] White, C. R., Alton, L. A., Bywater, C. L., Lombardi, E. J. and Marshall, D. J. (2022). Metabolic scaling is the product of life-history optimization. *Science* 377, 834-839. 10.1126/science.abm7649 35981018

[JEB246674C108] Zhou, J., Chen, L., Li, J., Li, H., Hong, Z., Xie, M., Chen, S., Yao, B. and Drevet, J. R. (2015). The semen pH affects sperm motility and capacitation. *PLoS One* 10, 1-15. 10.1371/journal.pone.0132974 PMC4501804 26173069

