# Supplementary material for: Per capita sperm metabolism is density dependent
Source: J Exp Biol. 2024 Mar 18;227(6):jeb246674. doi: 10.1242/jeb.246674 (PMC11006396; doi:10.1242/jeb.246674)
Supplement: Supplementary information [file jexbio-227-246674-s1.pdf]

Supplementary Materials and Methods

Literature searches and study selection

We used Google Scholar and Web of Science as our main information sources and searched their core collection. We developed specific search strings that allowed us to obtain studies that measured sperm metabolism under different sperm densities. Search terms were modified for each database because certain databases (i.e., Web of Science) provided less relevant results with more standard search terms and needed more directive terms to isolate the studies we were interested in. Google scholar has a broader selection of publication types and we had no issue finding relevant studies with standard search terms.

Search strings were modified for each database, as follows:

Google scholar:
(“sperm* oxygen consumption”), (“sperm oxygen consumption”), (“sperm oxygen consumption rate”), (“sperm OCR”), (“sperm aerobic metabolism”), (“sperm metabolism”), (“sperm respiration”), (“sperm concentration respiration”), (spermatozoa oxygen consumption sperm metabolism -human -bull -bulls -bovine -fowl -cock -boar -chicken -ram -rams -rat -rabbit -rats -stallion -stallions -clinical), (allintitle: sperm oxygen consumption), (allintitle: sperm concentration respiration, sperm "oxygen consumption" [limited to 2015-2022]), (Sperm* mitochondrial respiration oxygen consumption)

ISI Web of Science:
(Sperm oxygen consumption (Topic) and **"oxygen consumption"** (Abstract) not **embryos** (Topic) not **Sertoli** (All Fields) and **Sperm*** (Abstract)), (**Sperm oxygen consumption** (Topic) or **oxidative phosphorylation** (Topic) and **"oxygen consumption"** (Abstract) not **embryos** (Topic) not **Sertoli** (All Fields)), (**Sperm oxidative phosphorylation** (Topic) or **OXPHOS** (Topic) and **"oxygen consumption"** (Abstract)not **embryos** (Topic) not **Sertoli** (All Fields)), (**Sperm** (Topic) and **"mitochondrial oxygen consumption"** (Topic) not **embryos** (Topic) not **Sertoli**(All Fields)), (**Sperm*** (Topic) and **oxygen consumption rate** (Topic) and **OCR** (All Fields)),

Journal of Experimental Biology • Supplementary information

(Sperm* (Topic) and **mitochondrial respiration** (Topic)),
(Sperm* (Topic) and **"mitochondrial respiration"** (Topic) or **OXPHOS** (Topic)),
(**Sperm*** (Topic) and **"mitochondrial respiration"** (Topic) or **OXPHOS** (Topic) and **oxygen consumption** (Topic)),
(**Sperm*** (Topic) and **"mitochondrial respiration"** (Topic) or **OXPHOS** (Topic) and **oxygen consumption** (Topic) not **embryos** (Topic) not **Sertoli** (All Fields))

These searches found 3687 unique studies. The number of studies was reduced to 303 by AEP through examining the titles. Titles, abstracts and keywords were screened by AEP and uploaded into Rayyan (Ouzzani *et al.* 2016). The full-text of 303 studies where screened and excluded if they did not meet the inclusion criteria. A total of 234 studies were excluded. Eighty-eight studies were included in the final dataset.

Eligibility criteria

We focused on studies that measured the aerobic metabolism of sperm at different densities, across species. We selected studies based on six eligibility criteria (Table S3). First, we only included studies that measured sperm metabolism as oxygen consumption over time (e.g., VO_2 or $\mu l\ O_2$ sperm concentration^{-1} h^{-1}) and could be converted into common units. This excluded studies that used other units to measure metabolism (relative fluorescence units [RFU], NAD(P)H and FAD). We recognize that overlooking these metabolic parameters may have introduced bias in our dataset, however, oxygen consumption is one of the most widely reported metrics for sperm metabolism which allows us to compare measurements across many studies. Second, we focused on studies that presented a measurement of metabolic rate under control conditions – without any inhibitors or uncouplers. Third, we only considered studies that reported the actual sperm density (sperm ml^{-1}). If studies did not provide the actual sperm density but provided the chamber volume (ml) and sperm cells per chamber, they were also included. Fourth, we only included studies that reported ambient temperature (ºC) – ambient body temperature for endotherms and ambient environmental temperature for ectotherms – used during the metabolic measurements. Fifth, we included studies that presented the data in a way that could be extracted readily (table, plot, in-text) and provided summary statistics (mean, standard deviation, standard error). Sixth, methods were clear and could be followed – sperm handling

Journal of Experimental Biology • Supplementary information

procedures, extraction methods and diluent used were reported. If the studies did not meet these criteria, they were excluded from the dataset. Further details about inclusion criteria are presented in the supplemental (Table S3).

A total of 9558 studies were assessed for eligibility by "reviewer 1" and 5871 were rejected based on irrelevance (Fig. S1). Eighteen studies were not accessible to the authors. Titles, abstracts and keywords were screened by AEP, using Rayyan QCRI (Ouzzani *et al.* 2016) (Table S3). A total of 303 studies were screened by AEP for inclusion in this database and data from 234 studies did not follow the inclusion criteria. Search methods are further summarized in our PRISMA flowchart (Fig. S1).

Data extraction and effect size

We collected mean oxygen consumption data across densities along with sample size and measures of dispersion (i.e., standard deviation and standard error). Data was extracted by AEP and further checked by AEP and DJM. The data was primarily collected from original sources because there were few compilations (Jones & Murdoch 1996) that provided information on metabolism in sperm for a range of species that were also explicit with regards to concentration. Data that was extracted from tables or in text were directly input into the database. Data collected from figures were digitized using Webplotdigitizer [v. 4.5] (Rohatgi 2020). If data was missing from a source that was from 2005 or newer, we reached out to the authors. All estimates of sperm metabolism were measured *in vitro* (i.e., with a respirometer) regardless of fertilization mode (internal or external). Sperm were diluted into different densities with either sea water or activating medium (external fertilizers) or diluent media (internal fertilizers) (Table S1). We also included unpublished data for two species collected by the authors. Species were categorized by thermoregulation (endotherm and ectotherm) which is synonymous with fertilization mode (i.e., internal fertilizers = endotherms, external fertilizers = ectotherms [except for *Apis mellifera* and *Loligo pealei* – both ectotherms with internal fertilization]) in our dataset. Diluents were categorized as carbohydrate-free (i.e., contains no sugar) or carbohydrate-containing (i.e., contains sugar) media.

Other sperm handling methods (i.e., extraction, handling, diluent type) were mentioned in the results section and categorized in Table S1. Extraction methods included ejaculation or collected from the female reproductive tract. Sperm handling methods included if the sperm was used straight after extraction (fresh) or if the sperm was stored in a frozen or

Journal of Experimental Biology • Supplementary information

cooled state. Diluent type was categorized as either an activator (i.e., caused sperm to become active/motile or undergo capacitation) or extender (i.e., keep sperm inactive to extend longevity).

We followed a traditional meta-analytic approach to help build our database but used comparative analyses to test our hypothesis. We used this ‘hybrid’ approach because we were interested in estimating the biological scaling relationship between sperm concentration and metabolism. It is not possible to estimate the effect sizes and then analyse their scaling because the response variable is partly biological and partly statistical. Traditional meta-analyses calculate formal error and effect size for each study. We cannot scale our data by their error because doing so would remove the quantitative relationship that we are interested in – how metabolism covaries with density. We have included an analysis that uses log response ratio to provide an effect size index (in-text). The studies within our dataset have sample sizes that range from 2 to 176 (mostly between 4-6 replicates). We re-ran our analyses with studies that had 4 replicates or more and found no qualitative difference in our results to those that included the entire dataset (in-text).

Deviations from registration

We followed our original plans and procedures and did not deviate from them.

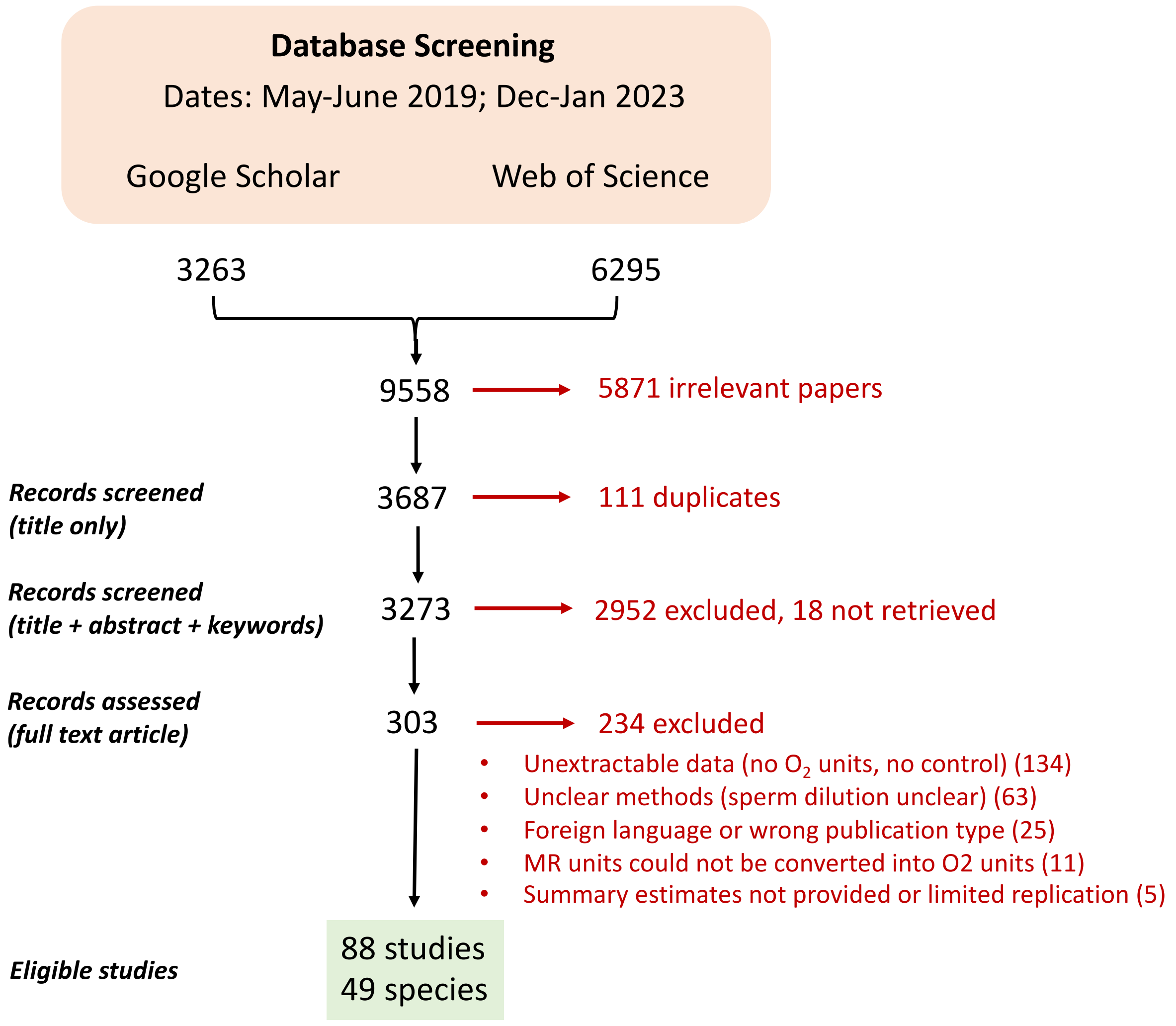

Fig. S1. PRIMSA flow chart. Summarizing the search methods, number of studies excluded and reasons for exclusions.

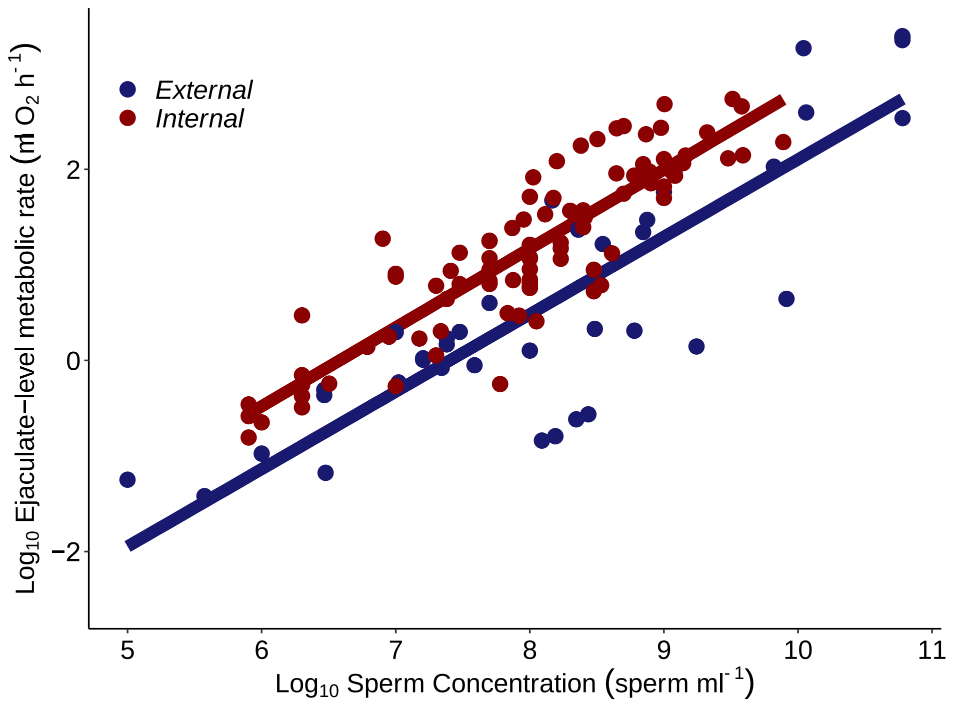

Fig. S2. Density-dependent Metabolism and Fertilization Mode. Plot shows the relationship between $\log_{10}$ ejaculate-level metabolic rate (μl O_2 h^{-1}) and $\log_{10}$ sperm concentration. Fitted lines represent line of best fit based on a linear mixed-effects model using all the data in the dataset. Each point represents an observation from a single species for 49 species. Colours indicate different fertilization modes (Internal [Red; N = 126] and External [Blue; N = 72]).

Journal of Experimental Biology • Supplementary information

Table S1. Density-dependent sperm metabolism data. Table showing ZO_2 (standardised ejaculate-level metabolic rate that was transformed linearly [µl O_2 conc^{-1} h^{-1}]), ZO_2 Conc (standardised concentration of sperm used for linear transformation [sperm ml^{-1}]), VO_2 (ejaculate-level metabolic rate measured during experiment [µl O_2 conc^{-1} h^{-1}]),VO_2 conc (sperm concentration used during experiment [sperm ml^{-1}]), Temp is temperature at which sperm metabolism was measured (ºC), Handling (cooled = sperm put on ice temporarily before use, fresh = sperm used immediately after extraction, frozen/thawed = sperm cryopreserved then thawed for use), Diluent used to dilute the sperm into different concentrations (BWW = Biggers-Whitten-Whittingham; KRP = calcium-free Krebs-Ringer phosphate; AM = activating medium; SW = seawater; SBB = Sodium bicarbonate buffer; SF = Seminal fluid; MCM = Minimum cultured media; PPB = Potassium phosphate buffer; BM = buffered medium; ISS = Isotonic salt solution; CM = Capacitating medium; PSS = physiological salt solution; DBT = De Boers solution; NKML = NaC1, 110 mM; KC1, 5 mM; MgSO, 2 mM), Diluent type (Activation = diluent caused sperm to activate, capacitate or become motile; Extender = diluent used to extend life of sperm), Extraction method (Ejaculated = sperm is collected by ejaculation; Extracted (M) = sperm is extracted from the male [testes, reproductive organs]; Extracted (F) = sperm is extracted from the female reproductive tract).

Taxon	ZO2 Conc	ZO2	VO2	VO2 Conc	Temp	Handling	Diluent	Diluent type	Extraction Method	Reference
Annelida										
Polychaeta (class):										
Arenicola marina			0.11	1.00E+06	14.5	Cooled	SW	Activation	Ejaculated	Campbell et al. (2017)
Arenicola marina	1.00E+08	7.98	3.99	5.00E+07	15	Fresh	SW	Activation	Extracted (M)	Pacey & Bentley (1993)
Galeolaria caespitosa			1.27	1.00E+08	21	Fresh	SW	Activation	Ejaculated	Kupriyanova & Havenhand (2005)
Galeolaria caespitosa			0.14	3.19E+06	21	Fresh	SW	Activation	Ejaculated	This study (unpub)
Galeolaria caespitosa			1.01	1.60E+07	21	Fresh	SW	Activation	Ejaculated	This study (unpub)
Galeolaria caespitosa			1.05	1.60E+07	21	Fresh	SW	Activation	Ejaculated	This study (unpub)
Galeolaria caespitosa			0.90	1.00E+08	21	Fresh	SW	Activation	Ejaculated	This study (unpub)
Galeolaria caespitosa			1.30	1.00E+07	21	Fresh	SW	Activation	Ejaculated	This study (unpub)
Galeolaria caespitosa			1.70	2.40E+07	21	Fresh	SW	Activation	Ejaculated	This study (unpub)
Galeolaria caespitosa			1.40	2.40E+07	21	Fresh	SW	Activation	Ejaculated	This study (unpub)
Urechis unicinctus	1.00E+09	50.27	100.53	2.00E+09	20	Fresh	SW	Activation	Ejaculated	Fujiwara et al. (1999)
Arthropoda										

Insecta (class):											
	Apis mellifera	1.00E+08	2.48	192.45	3.88E+09	32	Fresh	SF	Extender	Ejaculated	Verma & Shuel (1973)
	Apis mellifera	1.00E+08	3.61	140.07	7.76E+09	32	Fresh	SF	Extender	Ejaculated	Verma & Shuel (1973)
Malacostraca (class):											
	Metopograpsus messor			0.15	1.23E+08	30	Fresh	SW	Activation	Extracted (F)	Anilkumar et al (1996)
	Metopograpsus messor			0.16	1.55E+08	30	Fresh	SW	Activation	Extracted (F)	Anilkumar et al (1996)
	Metopograpsus messor			0.24	2.22E+08	30	Fresh	SW	Activation	Extracted (F)	Anilkumar et al (1996)
	Metopograpsus messor			0.27	2.73E+08	30	Fresh	SW	Activation	Extracted (F)	Anilkumar et al (1996)
Chordata											
Actinopterygii (class):											
	Acipenser baerii	1.00E+09	16.13	106.44	6.60E+09	15	Cooled	AM	Activation	Extracted (M)	Rahi et al. (2020)
	Alburnus chalcoides			393.12	1.15E+10	15	Fresh	AM	Activation	Ejaculated	Lahnsteiner et al. (1999)
	Chelon dumerili	1.00E+08	0.08	1.40	1.75E+09	22	Fresh	SW	Activation	Extracted (M)	Van Der Horst (1986)
	Chelon dumerili	1.00E+08	0.34	2.05	6.02E+08	22	Fresh	SW	Activation	Extracted (M)	Van Der Horst (1986)
	Chelon dumerili	1.00E+08	0.70	2.14	3.05E+08	22	Fresh	SW	Activation	Extracted (M)	Van Der Horst (1986)
	Clarias gariepinus	2.00E+09	1.07	4.41	8.20E+09	28	Fresh	Tris buffer	Activation	Extracted (M)	Mansour et al (2003)
	Cyprinus carpio	1.00E+09	6.33	4.71	6.02E+10	22	Fresh	AM	Activation	Extracted (M)	Boryshpolets et al (2008)
	Oncorhynchus mykiss	1.00E+10	17.00	45.02	2.65E+10	25	Fresh	AM	Activation	Extracted (M)	Terner and Korsh (1963)
	Oncorhynchus mykiss	1.00E+10	20.00	63.08	3.15E+10	25	Fresh	AM	Activation	Extracted (M)	Terner and Korsh (1963)
	Scophthalmus maximus			1845.04	1.10E+10	25	Frozen-thawed	AM	Activation	Extracted (M)	Dreanno et al (1999)
	Zosterisessor ophiocephalus	1.00E+06	0.15	0.43	2.93E+06	20	Fresh	AM	Activation	Extracted (M)	Locatello et al. (2020)
	Zosterisessor ophiocephalus	1.00E+06	0.17	0.49	2.93E+06	20	Fresh	AM	Activation	Extracted (M)	Locatello et al. (2020)
Amphibia (class):											

Journal of Experimental Biology • Supplementary information

	Rhinella arenarum	1.00E+08	3.27	3.31	1.01E+08	30	Fresh	KRP	Extender	Extracted (M)	Del Rio (1979)
	Xenopus laevis	1.00E+08	2.30	0.89	5.10E+06	22	Cooled	DBT	Extender	Extracted (M)	Bernardini et al (1988)
	Xenopus laevis			0.54	1.05E+07	22	Fresh	SW	Activation	Extracted (M)	Van Der Horst (1986)
	Xenopus laevis	1.00E+08	5.60	0.59	2.20E+07	22	Fresh	SW	Activation	Extracted (M)	Van Der Horst (1986)
	Xenopus laevis	1.00E+08	3.80	0.84	3.87E+07	22	Fresh	SW	Activation	Extracted (M)	Van Der Horst (1986)
Ascidiacea (class):											
	Ciona intestinalis			2370.82	6.00E+10	16	Fresh	SW	Activation	Extracted (M)	Brokaw and Benedict (1968)
	Ciona intestinalis			2395.01	6.00E+10	16	Fresh	SW	Activation	Extracted (M)	Brokaw and Benedict (1968)
	Ciona intestinalis			2467.58	6.00E+10	16	Fresh	SW	Activation	Extracted (M)	Brokaw and Benedict (1968)
	Ciona intestinalis	1.00E+09	54.03	108.06	6.00E+10	16	Fresh	SW	Activation	Extracted (M)	Brokaw and Benedict (1968)
	Ciona intestinalis			2249.86	2.00E+09	20	Fresh	SW	Activation	Ejaculated	Fujiwara et al. (1999)
Aves (class):											
	Gallus gallus	1.00E+08	5.00	50.00	5.00E+08	41	Fresh	SBB	Activation	Ejaculated	Ashizawa et al. (1985)
	Gallus gallus	1.00E+08	2.05	42.03	1.00E+09	40	Fresh	SBB	Activation	Ejaculated	Ashizawa et al. (1985)
	Gallus gallus	1.00E+08	2.96	8.88	1.00E+09	40	Fresh	NaCl + TES buffer	Extender	Ejaculated	Ashizawa et al. (1985)
	Gallus gallus	1.00E+09	46.70	233.50	3.25E+09	40	Fresh	NaCl + TES buffer	Extender	Ejaculated	Chaudhuri & Wishart (1988)
	Gallus gallus	1.00E+09	99.00	24.75	2.50E+08	41	Fresh	NaCl + TES buffer	Extender	Ejaculated	Froman & Kirby (2005)
	Gallus gallus	1.00E+10	660.00	66.00	2.50E+08	41	Fresh	NaCl + TES buffer	Extender	Ejaculated	Froman et al. (1999)
	Gallus gallus			37.20	5.00E+09	37	Fresh	ISS	Extender	Ejaculated	Schindler and Lehrer (1968)
	Gallus gallus	1.00E+08	11.10	55.50	2.05E+09	38	Fresh	KRP	Extender	Ejaculated	Scott et al (1962)
	Gallus gallus	1.00E+09	167.41	544.08	3.00E+08	41	Fresh	PPB	Activation	Ejaculated	Sexton (1974)
	Meleagris gallopavo	1.00E+06	0.07	78.62	1.15E+09	37	Fresh	Earle's Solution	Extender	Ejaculated	Bade et al. 1956)
	Meleagris gallopavo	1.00E+06	0.10	9.81	3.00E+08	41	Fresh	PPB	Activation	Ejaculated	Sexton (1974)
Mammalia (class):											

Journal of Experimental Biology • Supplementary information

Bos taurus			0.35	9.50E+08	37	Fresh	BM	Extender	Ejaculated	Al-Taha & Strzezek (1982)
Bos taurus	1.00E+09	71.00	85.63	1.21E+09	37	Fresh	Undiluted	Extender	Ejaculated	Bishop & Salisbury (1955)
Bos taurus	1.00E+09	84.00	95.59	1.14E+09	37	Fresh	Undiluted	Extender	Ejaculated	Bishop & Salisbury (1955)
Bos taurus	1.00E+09	91.00	106.47	1.17E+09	37	Fresh	Undiluted	Extender	Ejaculated	Bishop & Salisbury (1955)
Bos taurus	1.00E+09	96.00	139.30	1.45E+09	37	Fresh	Undiluted	Extender	Ejaculated	Bishop & Salisbury (1955)
Bos taurus	1.00E+08	35.80	17.90	1.00E+08	37	Fresh	Tris buffer	Activation	Ejaculated	Casas et al (2016)
Bos taurus	1.00E+08	7.40	61.42	1.00E+08	38	Frozen-thawed	Tris buffer	Activation	Ejaculated	Fernández & Córdoba (2014)
Bos taurus	1.00E+08	9.00	67.50	1.00E+08	38	Frozen-thawed	Tris buffer	Activation	Ejaculated	Fernández & Córdoba (2014)
Bos taurus	1.00E+08	16.13	112.93	1.00E+08	38	Frozen-thawed	Tyrodes Medium	Activation	Ejaculated	Fernández & Córdoba (2016)
Bos taurus	1.00E+08	18.40	36.80	1.00E+08	38	Frozen-thawed	Tyrodes Medium	Activation	Ejaculated	Fernández & Córdoba (2016)
Bos taurus	1.00E+08	0.42	0.63	1.00E+08	38	Frozen-thawed	Tyrodes Medium	Activation	Ejaculated	Fernández & Córdoba (2016)
Bos taurus	1.00E+08	0.87	1.31	5.00E+07	39	Fresh	Eqcellsire + Optiprep	Separator	Ejaculated	Garrett et al. (2008)
Bos taurus	1.00E+08	12.62	6.31	5.00E+07	39	Fresh	Eqcellsire + Optiprep	Separator	Ejaculated	Garrett et al. (2008)
Bos taurus	1.00E+08	13.00	6.50	5.00E+07	39	Fresh	Eqcellsire + Optiprep	Separator	Ejaculated	Garrett et al. (2008)
Bos taurus	1.00E+08	13.62	6.81	5.00E+07	39	Fresh	Eqcellsire + Optiprep	Separator	Ejaculated	Garrett et al. (2008)
Bos taurus	1.00E+08	17.44	8.72	5.00E+07	39	Fresh	Eqcellsire + Optiprep	Separator	Ejaculated	Garrett et al. (2008)
Bos taurus	1.00E+08	17.80	8.90	5.00E+07	39	Fresh	Eqcellsire + Optiprep	Separator	Ejaculated	Garrett et al. (2008)
Bos taurus	1.00E+08	35.54	17.77	5.00E+07	39	Fresh	Eqcellsire + Optiprep	Separator	Ejaculated	Garrett et al. (2008)
Bos taurus			0.16	7.00E+08	37	Fresh	KRP	Extender	Ejaculated	Lardy & Phillips (1942)
Bos taurus			9.00	8.00E+05	37	Frozen-thawed	Tyrodes Medium	Activation	Ejaculated	Magdanz et al (2019)
Bos taurus			6.47	8.00E+05	37	Frozen-thawed	Tyrodes Medium	Activation	Ejaculated	Magdanz et al (2019)
Bos taurus			11.75	8.00E+05	37	Frozen-thawed	Tyrodes Medium	Activation	Ejaculated	Magdanz et al (2019)
Bos taurus			5.73	8.30E+08	37	Fresh	KRP	Extender	Ejaculated	Murdoch & White (1966)
Bos taurus			5.96	7.50E+08	37	Fresh	KRP	Extender	Ejaculated	Murdoch & White (1966)
Bos taurus			7.02	2.00E+08	37	Fresh	KRP	Extender	Ejaculated	Norman et al (1958)
Bos taurus	1.00E+08	28.59	271.75	1.50E+08	30	Fresh	NKM	Extender	Ejaculated	Schoff (1995)

Bos taurus			0.26	1.23E+09	37	Fresh	KRP	Activation	Ejaculated	Scott et al (1962)
Bos taurus	1.00E+08	4.00	49.20	1.50E+08	37	Fresh	NKML	Activation	Ejaculated	Schoff and First (1995)
Canis familiaris	1.00E+08	6.50	16.58	1.75E+08	37	Fresh	KRP	Extender	Ejaculated	Murdoch & White (1966)
Canis familiaris	1.00E+08	11.43	26.28	2.55E+08	37	Fresh	KRP	Extender	Ejaculated	Murdoch & White (1966)
Canis familiaris	1.00E+08	4.10	7.18	2.30E+08	37	Fresh	KRP	Extender	Ejaculated	Scott et al (1962)
Cavia porcellus	1.00E+08	33.44	50.16	1.50E+08	37	Fresh	MCM	Extender	Extracted (M)	Rogers et al. (1979)
Equus asinus	1.00E+08	17.54	17.54	1.00E+08	37	Fresh	Saline solution	Extender	Ejaculated	Mann et al. (1963)
Equus asinus	1.00E+08	88.67	629.56	7.10E+08	37	Fresh	Saline solution	Extender	Ejaculated	Mann et al. (1963)
Equus asinus	1.00E+08	102.18	766.35	7.50E+08	37	Fresh	Saline solution	Extender	Ejaculated	Mann et al. (1963)
Equus caballus			104.29	1.00E+08	37	Fresh	Saline solution	Extender	Ejaculated	Mann et al. (1963)
Equus caballus	1.00E+08	17.08	17.08	6.30E+08	37	Fresh	Saline solution	Extender	Ejaculated	Mann et al. (1963)
Homo sapiens	1.00E+08	2.30	2.57	1.12E+08	37	Fresh	KRP	Extender	Ejaculated	Bernstein (1957)
Homo sapiens	1.00E+06	0.06	1.13	6.14E+06	37	Fresh	BWW Diluent	Activation	Ejaculated	Deutch et al. (1985)
Homo sapiens	1.00E+08	3.53	2.94	1.50E+07	37	Fresh	BWW Diluent	Activation	Ejaculated	Deutch et al. (1985)
Homo sapiens	1.00E+08	4.57	3.12	6.83E+07	37	Fresh	BWW Diluent	Activation	Ejaculated	Deutch et al. (1985)
Homo sapiens	1.00E+08	2.66	1.25	2.17E+07	37	Fresh	SF	Extender	Ejaculated	Deutch et al. (1985)
Homo sapiens	1.00E+08	2.12	4.22	8.90E+06	37	Fresh	SF	Extender	Ejaculated	Deutch et al. (1985)
Homo sapiens	1.00E+08		8.66	8.32E+07	37	Fresh	SF	Extender	Ejaculated	Deutch et al. (1985)
Homo sapiens			6.05	2.00E+07	36	Cooled	ISS	Extender	Ejaculated	Ferramosca et al (2012)
Homo sapiens			7.53	1.00E+07	37	Fresh	ISS	Extender	Ejaculated	Ferramosca et al (2021)
Homo sapiens			8.06	1.00E+07	37	Fresh	ISS	Extender	Ejaculated	Ferramosca et al (2021)
Homo sapiens	1.00E+08	11.28	1.69	2.57E+07	37	Fresh	KRP	Extender	Ejaculated	Ibrahim et al (1981)
Homo sapiens	1.00E+08	9.31	2.02	2.00E+07	37	Fresh	Ferticult medium	Activation	Ejaculated	Le Foll et al (2021)
Macaca fascicularis	1.00E+08	10.52	87.52	8.32E+08	35	Frozen-thawed	Extender	Extender	Ejaculated	Ackerman et al (1971)
Macaca mulatta	1.00E+08	14.72	193.94	1.32E+09	35	Frozen-thawed	Extender	Extender	Ejaculated	Ackerman et al (1971)
Macropus eugenii	1.00E+08	26.00	33.80	1.30E+08	37	Fresh	KRP	Extender	Ejaculated	Murdoch and Jones (1998)
Macropus eugenii			11.65	1.00E+08	34	Fresh	BM	Extender	Ejaculated	Murdoch et al.

Journal of Experimental Biology • Supplementary information

										(1999b)
Macropus eugenii			11.95	1.00E+08	34	Fresh	BM	Extender	Ejaculated	Murdoch et al. (1999b)
Macropus eugenii			16.20	1.00E+08	34	Fresh	BM	Extender	Ejaculated	Murdoch et al. (1999b)
Mus musculus	1.00E+08	20.93	6.28	1.20E+05	37	Fresh	Tyrodes Medium	Activation	Extracted (M)	Boell & Burkus (1984)
Mus musculus			0.01	1.48E+05	37	Fresh	KRP	Extender	Extracted (M)	Boell (1985)
Mus musculus			0.03	7.40E+04	37	Fresh	KRP	Extender	Extracted (M)	Boell (1985)
Mus musculus			0.08	3.37E+05	37	Fresh	KRP	Extender	Extracted (M)	Boell (1985)
Mus musculus	1.00E+06	0.06	0.01	5.00E+07	35	Fresh	PSS	Extender	Extracted (M)	Djakiew and Cardullo (1986)
Mus musculus	1.00E+08	23.50	11.75	2.00E+06	37	Fresh	Tris buffer	Activation	Ejaculated	Fang et al (2020)
Mus musculus			18.82	3.00E+07	37	Fresh	M2 medium	Activation	Extracted (M)	Miki et al (2004)
Mus musculus			0.32	2.00E+06	37	Fresh	Tyrodes Medium	Activation	Extracted (M)	Tourmente et al (2015)
Mus musculus			0.42	8.00E+06	37	Fresh	CM	Activation	Extracted (M)	Tourmente et al (2022)
Mus spicilegus			0.70	2.00E+06	37	Fresh	Tyrodes Medium	Activation	Extracted (M)	Tourmente et al (2015)
Mus spretus			0.55	2.00E+06	37	Fresh	Tyrodes Medium	Activation	Extracted (M)	Tourmente et al (2015)
Oryctolagus cuniculus	1.00E+08	6.20	19.22	5.00E+07	39	Fresh	KRP	Extender	Extracted (M)	Carter (1932)
Oryctolagus cuniculus	1.00E+07	0.09	0.57	6.00E+07	37	Fresh	Tyrodes Medium	Activation	Ejaculated	Castellini et al (2014)
Oryctolagus cuniculus			85.88	7.40E+07	37	Fresh	KRP	Extender	Ejaculated	Foley & Williams (1967)
Oryctolagus cuniculus	1.00E+08	7.34	3.67	1.70E+08	37	Fresh	KRP	Extender	Ejaculated	Hamner & Williams (1963)
Oryctolagus cuniculus	1.00E+08	1.80	6.12	6.00E+08	37	Fresh	KRP	Extender	Ejaculated	Killian et al. (1985)
Oryctolagus cuniculus	1.00E+08	6.80	11.56	3.40E+08	37	Fresh	KRP	Extender	Ejaculated	Murdoch & White (1966)
Oryctolagus cuniculus	1.00E+08	3.20	13.20	4.10E+08	37	Fresh	KRP	Extender	Ejaculated	Murdoch & White (1966)
Oryctolagus cuniculus	1.00E+08	32.90	24.28	3.10E+08	37	Fresh	KRP	Extender	Ejaculated	Murdoch & White (1968)
Ovis aries			12.60	7.40E+09	37	Fresh	BM	Extender	Ejaculated	Amir & Schindler (1967)
Ovis aries			51.60	1.06E+09	37	Fresh	BM	Extender	Ejaculated	Amir & Schindler (1967)
Ovis aries	1.00E+08	14.80	29.60	2.10E+09	37	Fresh	BM	Extender	Ejaculated	Amir & Schindler (1967)

Ovis aries	1.00E+08	33.18	29.86	3.80E+09	37	Fresh	BM	Extender	Ejaculated	Amir & Schindler (1967)
Ovis aries	1.00E+08	12.10	94.38	1.00E+08	37	Fresh	KRP	Extender	Ejaculated	Black et al. (1968)
Ovis aries	1.00E+08	12.80	128.00	9.00E+07	37	Fresh	KRP	Extender	Ejaculated	Foley & Williams (1967)
Ovis aries	1.00E+08	8.45	92.95	7.80E+08	37	Fresh	KRP	Extender	Ejaculated	Murdoch & White (1966)
Ovis aries	1.00E+09	88.40	654.16	1.00E+09	37	Fresh	KRP	Extender	Ejaculated	Murdoch & White (1966)
Ovis aries	1.00E+08	23.60	47.20	1.10E+09	37	Fresh	KRP	Extender	Ejaculated	Scott et al (1962)
Ovis aries	1.00E+09	119.90	455.62	1.00E+08	30	Fresh	Tris Buffer	Activation	Ejaculated	Simpson et al. (1987)
Ovis aries	1.00E+09	107.60	114.06	2.00E+08	37	Fresh	KRP	Extender	Extracted (M)	Voglmayr et al. (1970)
Ovis aries	1.00E+09	115.84	243.26	2.00E+08	37	Frozen-thawed	KRP	Extender	Extracted (M)	Voglmayr et al. (1970)
Ovis canadensis	1.00E+09	186.98	85.79	4.70E+08	37	Fresh	SF	Extender	Ejaculated	Winchester & McKenzie (1941)
Ovis canadensis	1.00E+09	93.90	115.97	1.39E+09	37	Fresh	SF	Extender	Ejaculated	Winchester & McKenzie (1941)
Ovis canadensis	1.00E+09	48.31	130.15	3.01E+09	37	Fresh	SF	Extender	Ejaculated	Winchester & McKenzie (1941)
Rattus norvegicus			0.47	1.06E+08	35	Fresh	PSS	Extender	Extracted (M)	Cardullo and Cone (1986)
Rattus norvegicus	1.00E+07	14.78	2.96	1.59E+08	35	Fresh	PSS	Extender	Extracted (M)	Cardullo and Cone (1986)
Rattus norvegicus	1.00E+08	9.22	6.92	2.40E+08	35	Fresh	PSS	Extender	Extracted (M)	Cardullo and Cone (1986)
Rattus norvegicus	1.00E+08	20.50	90.61	3.19E+08	35	Fresh	PSS	Extender	Extracted (M)	Cardullo and Cone (1986)
Rattus norvegicus	1.00E+08	77.98	82.43	7.35E+08	35	Fresh	PSS	Extender	Extracted (M)	Cardullo and Cone (1986)
Rattus norvegicus	1.00E+08	76.25	121.50	4.44E+08	35	Fresh	PSS	Extender	Extracted (M)	Cardullo and Cone (1986)
Rattus norvegicus	1.00E+08	73.58	176.80	5.01E+08	35	Fresh	PSS	Extender	Extracted (M)	Cardullo and Cone (1986)
Rattus norvegicus	1.00E+08	65.03	207.15	1.01E+09	35	Fresh	PSS	Extender	Extracted (M)	Cardullo and Cone (1986)
Rattus norvegicus			0.54	4.42E+08	37	Fresh	HEPES	Activation	Extracted (M)	Frenkel et al. (1978)
Rattus norvegicus	1.00E+08	31.62	232.52	2.00E+06	37	Fresh	KRP	Extender	Extracted (M)	Mitra and Chowdury (1992)
Rattus norvegicus	1.00E+08	60.46	268.60	7.50E+07	34	Fresh	KRP	Extender	Extracted (M)	Murdoch et al. (1999a)
Rattus norvegicus	1.00E+08	47.34	480.26	1.00E+07	36	Fresh	KRB	Extender	Extracted (M)	Yamashiro et al (2010)
Rattus norvegicus	1.00E+08	56.47	283.15	1.00E+07	37	Fresh	KRB	Extender	Extracted (M)	Yamashiro et al (2010)

Sus scrofa	1.00E+08	25.33	14.90	1.2E+09	37	Fresh	KRP	Extender	Ejaculated	Grotjan et al. (1975)
Sus scrofa	1.00E+08	29.07	17.10	9.0E+08	37	Fresh	KRP	Extender	Ejaculated	Grotjan et al. (1975)
Sus scrofa			0.22	1.0E+06	38	Fresh	Androhep	Activation	Ejaculated	Nesci et al (2020)
Sus scrofa	1.00E+09	115.71	47.08	4.1E+08	37	Fresh	SF	Extender	Ejaculated	Winchester & McKenzie (1941)
Sus scrofa	1.00E+09	109.94	63.63	2.1E+08	37	Fresh	SF	Extender	Ejaculated	Winchester & McKenzie (1941)
Sus scrofa	1.00E+09	100.42	76.00	3.1E+08	37	Fresh	SF	Extender	Ejaculated	Winchester & McKenzie (1941)
Sus scrofa	1.00E+09	28.34	78.85	4.1E+08	37	Fresh	SF	Extender	Ejaculated	Winchester & McKenzie (1941)
Sus scrofa	1.00E+09	75.95	90.10	5.8E+08	37	Fresh	SF	Extender	Ejaculated	Winchester & McKenzie (1941)
Sus scrofa	1.00E+09	101.07	90.82	7.6E+08	37	Fresh	SF	Extender	Ejaculated	Winchester & McKenzie (1941)
Sus scrofa	1.00E+08	18.29	4.39	2.8E+09	37	Fresh	SF	Extender	Ejaculated	Foley & Williams (1967)
Sus scrofa	1.00E+09	122.08	31.48	2.6E+08	37	Fresh	SF	Extender	Ejaculated	Winchester & McKenzie (1941)
Trichosurus vulpecula	1.00E+08	44.67	13.40	3.00E+07	37	Fresh	KRP	Extender	Ejaculated	Rodger & Suter (1978)
Vulpes fulva			0.57	3.19E+06	37	Fresh	SF	Extender	Ejaculated	Bishop (1942)
Echinodermata										
Echinoidae (class):										
Anthocidaris crassispina			4.82	1.00E+08	20	Fresh	SW	Activation	Ejaculated	Fujiwara et al. (1982)
Anthocidaris crassispina			5.09	1.00E+08	20	Fresh	SW	Activation	Ejaculated	Fujiwara et al. (1982)
Anthocidaris crassispina			5.13	1.00E+08	20	Fresh	SW	Activation	Ejaculated	Fujiwara et al. (1982)
Anthocidaris crassispina	1.00E+09	42.20	84.40	2.00E+09	20	Fresh	SW	Activation	Ejaculated	Fujiwara et al. (1999)
Arbacia lixula	1.00E+09		0.05	1.00E+07	20	Fresh	SW	Activation	Ejaculated	Mita et al. (1994)
Clypeaster japonicus			3.25	1.00E+08	20	Fresh	SW	Activation	Ejaculated	Fujiwara et al. (1982)
Clypeaster japonicus			3.41	1.00E+08	20	Fresh	SW	Activation	Ejaculated	Fujiwara et al. (1982)
Clypeaster japonicus			4.09	1.00E+08	20	Fresh	SW	Activation	Ejaculated	Fujiwara et al. (1982)
Echinus esculentus	1.12E+10	94.78	2.83	1.12E+09	15	Fresh	SW	Activation	Ejaculated	Rothschild & Tuft (1948)
Echinus esculentus	2.35E+10	144.67	3.93	6.79E+08	15	Fresh	SW	Activation	Ejaculated	Rothschild & Tuft (1948)

Journal of Experimental Biology • Supplementary information

Echinus esculentus	2.93E+10	134.60	5.15	3.36E+08	15	Fresh	SW		Activation	Ejaculated	Rothschild (1950)
Echinus esculentus	2.93E+10	251.33	5.82	6.41E+08	15	Fresh	SW		Activation	Ejaculated	Rothschild (1950)
Heliocidaris erythrogramma			0.04	3.75E+05	21	Fresh	SW		Activation	Ejaculated	This study (unpub)
Heliocidaris erythrogramma			0.07	3.00E+06	21	Fresh	SW		Activation	Ejaculated	This study (unpub)
Heliocidaris erythrogramma			1.97	1.00E+07	21	Fresh	SW		Activation	Ejaculated	This study (unpub)
Heliocidaris erythrogramma			1.99	3.00E+07	21	Fresh	SW		Activation	Ejaculated	This study (unpub)
Hemicentrotus pulcherrimus			5.51	1.00E+08	20	Fresh	SW		Activation	Ejaculated	Fujiwara et al. (1982)
Hemicentrotus pulcherrimus			5.58	1.00E+08	20	Fresh	SW		Activation	Ejaculated	Fujiwara et al. (1982)
Hemicentrotus pulcherrimus			5.58	1.00E+08	20	Fresh	SW		Activation	Ejaculated	Fujiwara et al. (1982)
Hemicentrotus pulcherrimus			24.19	2.00E+08	20	Fresh	SW		Activation	Ejaculated	Fujiwara et al. (1983)
Hemicentrotus pulcherrimus	1.00E+08	1.71	3.41	2.50E+09	20	Fresh	SW		Activation	Ejaculated	Mohri (1956)
Hemicentrotus pulcherrimus	1.00E+09	44.90	112.25	2.50E+09	20	Fresh	SW		Activation	Ejaculated	Mohri (1956)
Hemicentrotus pulcherrimus	1.00E+09	51.60	129.00	1.30E+09	17	Fresh	SW		Activation	Ejaculated	Ohtake (1976)
Paracentrotus lividus	7.00E+08	22.00	22.00	1.00E+07	20	Fresh	SW		Activation	Ejaculated	Mita et al. (1994)
Paracentrotus lividus	7.00E+08	33.00	16.50	7.00E+08	15	Fresh	SW		Activation	Ejaculated	Rothschild (1956)
Paracentrotus lividus	7.00E+08	71.00	23.33	3.50E+08	15	Fresh	SW		Activation	Ejaculated	Rothschild (1956)
Paracentrotus lividus	1.00E+09		0.09	2.30E+08	15	Fresh	SW		Activation	Ejaculated	Rothschild (1956)
Pseudocentrotus depressus			5.43	1.00E+08	20	Fresh	SW		Activation	Ejaculated	Fujiwara et al. (1982)
Pseudocentrotus depressus			28.96	1.00E+08	20	Fresh	SW		Activation	Ejaculated	Fujiwara et al. (1982)
Pseudocentrotus depressus			52.35	1.00E+08	20	Fresh	SW		Activation	Ejaculated	Fujiwara et al. (1982)
Pseudocentrotus depressus	1.00E+08	32.20	47.23	1.47E+08	20	Cooled	SW		Activation	Ejaculated	Kazama and Hino (2012)
Pseudocentrotus depressus			4.70	1.00E+05	20	Fresh	SW		Activation	Ejaculated	Kazama et al (2006)
Pseudocentrotus depressus			111.38	1.46E+08	18	Fresh	SW		Activation	Ejaculated	Ohtake (1976)
Pseudocentrotus depressus			186.01	2.60E+08	18	Fresh	SW		Activation	Ejaculated	Ohtake (1976)
Pseudocentrotus depressus			301.84	7.29E+08	18	Fresh	SW		Activation	Ejaculated	Ohtake (1976)
Pseudocentrotus depressus	1.00E+08	5.11	10.21	1.40E+09	18	Fresh	SW		Activation	Ejaculated	Ohtake (1976)
Pseudocentrotus depressus	1.00E+07	5.64	0.06	3.16E+09	18	Fresh	SW		Activation	Ejaculated	Ohtake (1976)
Pseudocentrotus depressus			5.00	2.00E+08	20	Fresh	SW		Activation	Ejaculated	Yasumasu et al.

											(1980)
	Strongylocentrotus intermedius			5.74	1.00E+08	20	Fresh	SW	Activation	Ejaculated	Fujiwara et al. (1982)
	Toxopneustes pileolus			4.31	1.00E+08	20	Fresh	SW	Activation	Ejaculated	Fujiwara et al. (1982)
	Toxopneustes pileolus			4.72	1.00E+08	20	Fresh	SW	Activation	Ejaculated	Fujiwara et al. (1982)
	Toxopneustes pileolus			4.81	1.00E+08	20	Fresh	SW	Activation	Ejaculated	Fujiwara et al. (1982)
Mollusca											
Bivalvia (class):											
	Crassostrea gigas	1.00E+09	47.85	95.69	2.00E+09	20	Fresh	SW	Activation	Ejaculated	Fujiwara et al. (1999)
	Pinctada margaritifera	1.00E+09	39.42	29.56	1.00E+09	25	Fresh	SW	Activation	Extracted (M)	Demoy-Schneider et al (2018)
	Pinctada margaritifera			57.79	7.50E+08	25	Fresh	SW	Activation	Extracted (M)	Demoy-Schneider et al. (2012)
Cephalopoda (class):											
	Loligo pealei			71.41	8.00E+08	20	Fresh	SW	Activation	Extracted (M)	Austin et al. (1964)

(Carter 1932; Winchester & McKenzie 1941; Bishop 1942; Lardy & Phillips 1942; Rothschild 1950, 1956; Rothschild & Tuft 1950; Bishop & Salisbury 1955; Bade *et al.* 1956; Mohri 1956; Bernstein 1957; Norman *et al.* 1958; Scott *et al.* 1962; Hamner & Williams 1963; Mann *et al.* 1963; Terner & Korsh 1963; Austin *et al.* 1964; Murdoch & White 1966, 1968; Amir & Schindler 1967; Foley & Williams 1967; Black *et al.* 1968; Brokaw & Benedict 1968; Schindler & Lehrer 1968; Voglmayr *et al.* 1970; Ackerman & Roussel 1971; Verma & Shuel 1973; Sexton 1974; Grotjan *et al.* 1975; Ohtake 1976; Frenkel *et al.* 1978; Rodger & Suter 1978; del Rio 1979; Rogers *et al.* 1979; Yasumasu *et al.* 1980; Ibrahim *et al.* 1981; Al-Taha & Strzezek 1982; Fujiwara *et al.* 1982, 1983, 1999; Boell & Burkus 1984; Ashizawa *et al.* 1985; Boell 1985; Deutch *et al.* 1985; Killian *et al.* 1985; Cardullo & Cone 1986; Djakiew & Cardullo 1986; van der Horst 1986; Simpson *et al.* 1987; Bernardini *et al.* 1988; Chaudhuri & Wishart 1988; Mitra & Chowdury 1992; Pacey & Bentley 1993; Mita *et al.* 1994; Schoff 1995; Schoff & First 1995; Anilkumar *et al.* 1996; Murdoch & Jones 1998; Dreanno *et al.* 1999b; Froman *et al.* 1999; Lahnsteiner *et al.* 1999a; Murdoch *et al.* 1999b, a; Mansour *et al.* 2003; Miki *et al.* 2004; Froman & Kirby 2005; Kupriyanova & Havenhand 2005; Kazama *et al.* 2006; Garrett *et al.* 2008; Boryshpolets *et al.* 2009; Yamashiro *et al.* 2010a, b; Ferramosca *et al.* 2012, 2021; Kazama & Hino 2012; Castellini *et al.* 2014; Fernández & Córdoba 2014, 2016; Tourmente *et al.* 2015, 2022; Casas *et al.* 2016; Campbell *et al.* 2017; Demoy-Schneider *et al.* 2018; Magdanz *et al.* 2019; Fang *et al.* 2020; Locatello *et al.* 2020; Nesci *et al.* 2020; Rahi *et al.* 2020; le Foll *et al.* 2021)

Journal of Experimental Biology • Supplementary information

Table S2. PRISMA Checklist

**Checklist Item**	**Sub-item Number**	**Sub-item**	**Reported by Authors?**	**Location**
Title and abstract	1.1	Identify the review as a systematic review, meta-analysis, or both	Yes	Title; Abstract
	1.2	Summarise the aims and scope of the review	Yes	Abstract
	1.3	Describe the data set	Yes	Abstract
	1.4	State the results of the primary outcome	Yes	Abstract
	1.5	State conclusions	Yes	Abstract
	1.6	State limitations	Yes	Abstract
Aims and questions	2.1	Provide a rationale for the review	Yes	Introduction
	2.2	Reference any previous reviews or meta-analyses on the topic	Yes	Introduction
	2.3	State the aims and scope of the review (including its generality)	Yes	Introduction
	2.4	State the primary questions the review addresses (e.g. which moderators were tested)	Yes	Introduction; Materials and Methods
	2.5	Describe whether effect sizes were derived from experimental and/or observational comparisons	NA	
Review registration	3.1	Register review aims, hypotheses (if applicable), and methods in a time-stamped and publicly accessible archive and provide a link to the registration in the methods section of the manuscript. Ideally registration occurs before the search, but it can be done at any stage before data analysis.	NA	
	3.2	Describe deviations from the registered aims and methods	Yes	Supplementary methods: *Deviations from registration*
	3.3	Justify deviations from the registered aims and methods	Yes	Supplementary methods: *Deviations from registration*
Eligibility criteria	4.1	Report the specific criteria used for including or excluding studies when screening titles and/or abstracts, and full texts, according to the aims of the systematic review (e.g. study design, taxa, data availability)	Yes	Materials and Methods: *Literature searches and study selection;*

Journal of Experimental Biology • Supplementary information

				Supplementary methods: *Eligibility criteria*
	4.2	Justify criteria, if necessary (i.e. not obvious from aims and scope)	Yes	Materials and Methods: *Literature searches and study selection;* Supplementary methods: *Eligibility criteria*
Finding studies	5.1	Define the type of search (e.g. comprehensive search, representative sample)	Yes	Materials and Methods: *Literature searches and study selection*
	5.2	State what sources of information were sought (e.g. published and unpublished studies, personal communications)	Yes	Materials and Methods: *Literature searches and study selection;* Supplementary methods: *Eligibility criteria*
	5.3	Include, for each database searched, the exact search strings used, with keyword combinations and Boolean operators	Yes	Supplementary methods: *Literature searches and study selection*
	5.4	Provide enough information to repeat the equivalent search (if possible), including the timespan covered (start and end dates)	Yes	Materials and Methods: *Literature searches and study selection;* Supplementary Methods: *Eligibility criteria*
Study selection	6.1	Describe how studies were selected for inclusion at each stage of the screening process (e.g. use of decision trees, screening software)	Yes	Materials and Methods: *Literature searches and study*

Journal of Experimental Biology • Supplementary information

				selection; Supplementary Methods*: Eligibility criteria*; Fig. S1
	6.2	Report the number of people involved and how they contributed (e.g. independent parallel screening)	Yes	Supplementary Methods: *Literature searches and study selection;* Supplementary Methods*: Eligibility criteria*
Data collection process	7.1	Describe where in the reports data were collected from (e.g. text or figures)	Yes	Materials and Methods: *Data extraction and effect size*
	7.2	Describe how data were collected (e.g. software used to digitize figures, external data sources)	Yes	Materials and Methods: *Data extraction and effect size*
	7.3	Describe moderator variables that were constructed from collected data (e.g. number of generations calculated from years and average generation time)	NA	
	7.4	Report how missing or ambiguous information was dealt with during data collection (e.g. authors of original studies were contacted for missing descriptive statistics, and/or effect sizes were calculated from test statistics)	Yes	Supplementary Methods: *Data extraction and effect size*
	7.5	Report who collected data	Yes	Supplementary Methods: *Eligibility criteria*
	7.6	State the number of extractions that were checked for accuracy by co-authors	Yes	Supplementary Methods: *Eligibility criteria*
Data items	8.1	Describe the key data sought from each study	Yes	Materials and Methods: *Data extraction*

Journal of Experimental Biology • Supplementary information

	8.2	Describe items that do not appear in the main results, or which could not be extracted due to insufficient information	Yes	Materials and Methods: *Data extraction*
	8.3	Describe main assumptions or simplifications that were made (e.g. categorising both 'length' and 'mass' as 'morphology')	NA: no assumptions needed to be made	
	8.4	Describe the type of replication unit (e.g. individuals, broods, study sites)	Yes	Materials and Methods: *Data extraction*
Assessment of individual study quality	9.1	Describe whether the quality of studies included in the systematic review or meta-analysis was assessed (e.g. blinded data collection, reporting quality, experimental vs. observational)	NA	
	9.2	Describe how information about study quality was incorporated into analyses (e.g. meta-regression and/or sensitivity analysis)	Yes	Materials and Methods: *Data extraction and effect size;* Supplementary Methods: *Data extraction and effect size*
Effect size measures	10.1	Describe effect size(s) used	Yes	Materials and Methods: *Data extraction and effect size;* Supplementary Methods: *Data extraction and effect size*
	10.2	Provide a reference to the equation of each calculated effect size (e.g. standardised mean difference, log response ratio) and (if applicable) its sampling variance	Yes	Materials and Methods: *Data extraction and effect size;* Supplementary Methods: *Data extraction and effect size*
	10.3	If no reference exists, derive the equations for each effect size and state the assumed sampling distribution(s)	NA	
Missing data	11.1	Describe any steps taken to deal with missing data during analysis (e.g. imputation, complete case, subset	Yes	Supplementary Methods: *Data extraction and*

Journal of Experimental Biology • Supplementary information

		analysis)		*effect size*
	11.2	Justify the decisions made to deal with missing data	Yes	Materials and Methods: *Data extraction*
Meta-analytic model description	12.1	Describe the models used for synthesis of effect sizes	Yes	Materials and Methods: *Data extraction and effect size;* Supplementary Methods: *Data extraction and effect size*
	12.2	The most common approach in ecology & evolution will be a random-effects model, often with a hierarchical/multilevel structure. If other types of models are chosen (e.g. common/fixed effects model, unweighted model), provide justification for this choice	Yes; mixed effects model with species as a random effect	Materials and Methods; Results
Software	13.1	Describe the statistical platform used for inference (e.g. *R*)	Yes	Materials and Methods: *Statistical Analyses*
	13.2	Describe the packages used to run models	Yes	Materials and Methods: *Statistical Analyses*
	13.3	Describe the functions used to run models	Yes	Materials and Methods: *Statistical Analyses*
	13.4	Describe any arguments that differed from the default settings	NA	
	13.5	Describe the version numbers of all software used	Yes	Materials and Methods: *Statistical Analyses*
Non-independence	14.1	Describe the types of non-independence encountered (e.g. phylogenetic, spatial, multiple measurements over time)	Yes	Materials and Methods: *Statistical Analyses*
	14.2	Describe how non-independence has been handled	Yes	Materials and Methods: *Statistical Analyses*
	14.3	Justify decisions made	Yes	Materials and Methods:

Journal of Experimental Biology • Supplementary information

				Statistical Analyses
Meta-regression and model selection	15.1	Provide a rationale for the inclusion of moderators (covariates) that were evaluated in meta-regression models	Yes	Materials and Methods: *Statistical Analyses*
	15.2	Justify the number of parameters estimated in models, in relation to the number of effect sizes and studies (e.g. interaction terms were not included due to insufficient sample sizes)	NA	
	15.3	Describe any process of model selection	Yes	Materials and Methods: *Statistical Analyses*
Publication bias and sensitivity analyses	16.1	Describe assessments of the risk of bias due to missing results (e.g. publication, time-lag, and taxonomic biases)	NA	
	16.2	Describe any steps taken to investigate the effects of such biases (if present)	NA	
	16.3	Describe any other analyses of robustness of the results, e.g. due to effect size choice, weighting or analytical model assumptions, inclusion or exclusion of subsets of the data, or the inclusion of alternative moderator variables in meta-regressions	Yes	Materials and Methods: *Data extraction and effect size;* Supplementary Methods: *Data extraction and effect size*
Clarification of post hoc analyses	17.1	When hypotheses were formulated after data analysis, this should be acknowledged.	Yes	Supplementary Methods: *Deviations from registration*
Metadata, data, and code	18.1	Share metadata (i.e. data descriptions)	Yes	Dryad
	18.2	Share data required to reproduce the results presented in the manuscript	Yes	Dryad
	18.3	Share additional data, including information that was not presented in the manuscript (e.g. raw data used to calculate effect sizes, descriptions of where data were located in papers)	Yes	Dryad
	18.4	Share analysis scripts (or, if a software package with graphical user interface (GUI) was used, then describe full model specification and	Yes	Dryad

Journal of Experimental Biology • Supplementary information

		fully specify choices)		
Results of study selection process	19.1	Report the number of studies screened	Yes	Materials and Methods: *Literature searches, study selection;* Supplementary Methods*: Eligibility criteria*; Fig. S1
	19.2	Report the number of studies excluded at each stage of screening	Yes	Supplementary Methods: *Eligibility criteria*; Fig. S1
	19.3	Report brief reasons for exclusion from the full text stage	Yes	Supplementary Methods: *Eligibility criteria*; Fig. S1
	19.4	Present a Preferred Reporting Items for Systematic Reviews and Meta-Analyses (PRISMA)-like flowchart (www.prisma-statement.org).	Yes	Fig. S1
Sample sizes and study characteristics	20.1	Report the number of studies and effect sizes for data included in meta-analyses	NA	
	20.2	Report the number of studies and effect sizes for subsets of data included in meta-regressions	NA	
	20.3	Provide a summary of key characteristics for reported outcomes (either in text or figures; e.g. one quarter of effect sizes reported for vertebrates and the rest invertebrates)	NA	
	20.4	Provide a summary of limitations of included moderators (e.g. collinearity and overlap between moderators)	NA	
	20.5	Provide a summary of characteristics related to individual study quality (risk of bias)	NA	
Meta-analysis	21.1	Provide a quantitative synthesis of results across studies, including estimates for the mean effect size, with confidence/credible intervals	NA	

Journal of Experimental Biology • Supplementary information

Heterogeneity	22.1	Report indicators of heterogeneity in the estimated effect (e.g. I2, tau2 and other variance components)	NA	
Meta-regression	23.1	Provide estimates of meta-regression slopes (i.e. regression coefficients) and confidence/credible intervals	Yes	Results
	23.2	Include estimates and confidence/credible intervals for all moderator variables that were assessed (i.e. complete reporting)	Yes	Results
	23.3	Report interactions, if they were included	Yes	Results
	23.4	Describe outcomes from model selection, if done (e.g. R2 and AIC)	Yes	Results; Supplemental Material
Outcomes of publication bias & sensitivity analyses	24.1	Provide results for the assessments of the risks of bias (e.g. Egger's regression, funnel plots)	NA	
	24.2	Provide results for the robustness of the review's results (e.g. subgroup analyses, meta-regression of study quality, results from alternative methods of analysis, and temporal trends)	NA	
Discussion	25.1	Summarise the main findings in terms of the magnitude of effect	Yes	Discussion
	25.2	Summarise the main findings in terms of the precision of effects (e.g. size of confidence intervals, statistical significance)	Yes	Discussion
	25.3	Summarise the main findings in terms of their heterogeneity	NA	
	25.4	Summarise the main findings in terms of their biological/practical relevance	Yes	Discussion
	25.5	Compare results with previous reviews on the topic, if available	Yes	Discussion
	25.6	Consider limitations and their influence on the generality of conclusions, such as gaps in the available evidence (e.g. taxonomic and geographical research biases)	Yes	Discussion
Contributions and funding	26.1	Provide names, affiliations, and funding sources of all co-authors	Yes	Title page
	26.2	List the contributions of each co-author	Yes	Authorship
	26.3	Provide contact details for the corresponding author	Yes	Title page
	26.4	Disclose any conflicts of interest	Yes	Conflicts of interest
References	27.1	Provide a reference list of all studies included in the systematic review or meta-analysis	Yes	Data sources (References)
	27.2	List included studies as referenced sources (e.g. rather than listing them in a table or supplement)	Yes	Data sources (References)

Journal of Experimental Biology • Supplementary information

Table S3. Description of the inclusion criteria used to screen full texts of studies.

	Description
**1**	Studies were included if they presented sperm metabolism as oxygen consumed over time (i.e., μl O_2 sperm concentration^{-1} h^{-1}) and could be converted into a common unit for comparison. If studies used other units that could not be readily converted into oxygen consumption units, they were excluded from the dataset.
**2**	Studies that measure sperm oxygen consumption under control conditions were included in the dataset. Studies that added a metabolic uncoupler or inhibitor and did not report a control, were excluded from the dataset.
**3**	Studies that reported the actual sperm density (i.e., density that sperm was diluted to for measuring metabolism) where included in the dataset. Studies that did not report the actual sperm density and only reported a 'standardized' sperm density (see Materials and Methods: Misestimation) were excluded from the dataset. If studies reported the number of sperm per chamber volume along with the chamber volume, those studies were included in the dataset.
**4**	Studies that reported the ambient temperature that sperm metabolism was measured at, were included in the dataset. If the study manipulated temperature (i.e., used temperature as a treatment), only the metabolic estimate for the control temperature was used.
**5**	The data needs to be accessible (i.e., table, in-text or figure) and easy to extract. Summary statistics (mean, standard deviation, standard error) need to be provided or easily calculated from the information reported.
**6**	Methods need to be clear and transparent. Sperm handling methods (fresh, frozen, cooled), extraction methods (ejaculated or extracted) and diluent used (seawater, Ringers solution, etc), all need to be reported.

Journal of Experimental Biology • Supplementary information

Table S4. Oxygen Saturation Data. This table contains information for oxygen saturation data for species in our dataset which reported chamber volume, temperature and duration over which sperm oxygen consumption was measured. If the study did not report one of these factors, they were omitted from this table. VO_2 (ml O_2 conc^{-1} h^{-1}) = oxygen consumption rate, VO2 Conc = sperm concentration (sperm ml^{-1}), Incubation (hours) = time over which VO_2 was taken, chamber volume (L), temperature (°C), solubility (ml O_2 L^{-1})(Cameron 1986), percent O_2 decline (calculated by: 1) Chamber volume x Solubility [N1], 2) VO_2 x Incubation [N2], 3) [N2/N1] x 100).

Phyla	Class	Species	VO2	VO2 Conc	Incubation	Chamber Volume	Temperature	Solubility	% O2 decline	Reference
Annelida	Polychaeta	*Arenicola marina*	1.06E-04	1.00E+06	0.33	4.50E-04	14.5	5.75	1.36	Campbell et al. (2017)
Annelida	Polychaeta	*Arenicola marina*	3.99E-03	5.00E+07	1.00	7.00E-03	15.0	5.75	9.91	Pacey & Bentley (1993)
Annelida	Polychaeta	*Galeolaria caespitosa*	1.27E-03	1.00E+08	0.33	2.00E-04	21.0	5.11	41.36	Kupriyanova & Havenhand (2005)
Annelida	Polychaeta	*Galeolaria caespitosa*	1.57E-04	2.40E+07	1.00	7.50E-04	21.0	5.11	4.09	This study (unpub)
Annelida	Polychaeta	*Galeolaria caespitosa*	1.01E-03	3.19E+06	1.00	7.50E-04	21.0	5.11	26.48	This study (unpub)
Annelida	Polychaeta	*Galeolaria caespitosa*	1.05E-03	1.60E+07	1.00	7.50E-04	21.0	5.11	27.49	This study (unpub)
Annelida	Polychaeta	*Galeolaria caespitosa*	1.06E-03	1.60E+07	1.00	7.50E-04	21.0	5.11	27.60	This study (unpub)
Annelida	Polychaeta	*Galeolaria caespitosa*	1.32E-03	1.00E+08	1.00	7.50E-04	21.0	5.11	34.50	This study (unpub)
Annelida	Polychaeta	*Galeolaria caespitosa*	1.55E-03	1.00E+07	1.00	7.50E-04	21.0	5.11	40.36	This study (unpub)
Annelida	Polychaeta	*Galeolaria caespitosa*	1.70E-03	2.40E+07	1.00	7.50E-04	21.0	5.11	44.42	This study (unpub)
Arthropoda	Malacostraca	*Metopograpsus messor*	1.45E-04	1.23E+08	1.00	2.50E-02	30.0	4.34	0.13	Anilkumar et al (1996)
Arthropoda	Malacostraca	*Metopograpsus messor*	1.61E-04	1.55E+08	1.00	2.50E-02	30.0	4.34	0.15	Anilkumar et al (1996)
Arthropoda	Malacostraca	*Metopograpsus messor*	2.42E-04	2.22E+08	1.00	2.50E-02	30.0	4.34	0.22	Anilkumar et al (1996)
Arthropoda	Malacostraca	*Metopograpsus messor*	2.73E-04	2.73E+08	1.00	2.50E-02	30.0	4.34	0.25	Anilkumar et al (1996)
Chordata	Actinopterygii	*Clarias gariepinus*	4.41E-03	8.20E+09	0.03	2.00E-05	28.0	4.49	163.54	Mansour et al (2003)
Chordata	Actinopterygii	*Cyprinus carpio*	4.71E-03	6.02E+10	0.07	1.00E-03	22.0	5.01	6.27	Boryshpolets et al (2008)
Chordata	Actinopterygii	*Salmo gairdnerii*	4.50E-02	2.65E+10	1.00	3.00E-03	25.0	4.74	316.57	Terner and Korsh (1963)
Chordata	Actinopterygii	*Salmo gairdnerii*	6.31E-02	3.15E+10	1.00	3.00E-03	25.0	4.74	443.60	Terner and Korsh (1963)
Chordata	Actinopterygii	*Zosterisessor ophiocephalus*	4.33E-04	2.93E+06	0.03	1.10E-04	20.0	5.21	2.52	Locatello et al. (2020)
Chordata	Actinopterygii	*Zosterisessor ophiocephalus*	4.92E-04	2.93E+06	0.03	1.10E-04	20.0	5.21	2.86	Locatello et al. (2020)
Chordata	Amphibia	*Rhinella arenarum*	3.14E-03	1.01E+08	0.33	1.60E-03	30.0	4.34	15.05	Del Rio (1979)
Chordata	Aves	*Gallus gallus*	3.72E-02	2.50E+08	0.05	2.00E-03	37.0	3.66	25.41	Froman et al. (1999)
Chordata	Aves	*Gallus gallus*	8.88E-03	3.00E+08	0.50	7.00E-03	41.0	3.66	17.33	Sexton (1974)

Chordata	Aves	*Meleagris gallopavo*	7.86E-02	1.15E+09	2.00	1.70E-02	37.0	3.84	240.88	Bade et al. (1956)
Chordata	Aves	*Meleagris gallopavo*	5.30E-03	3.00E+08	0.50	7.00E-03	41.0	3.66	10.33	Sexton (1974)
Chordata	Mammalia	*Bos taurus*	8.56E-02	1.21E+09	1.00	5.00E-02	37.0	3.84	44.60	Bishop & Salisbury (1955)
Chordata	Mammalia	*Bos taurus*	9.56E-02	1.14E+09	1.00	5.00E-02	37.0	3.84	49.79	Bishop & Salisbury (1955)
Chordata	Mammalia	*Bos taurus*	1.06E-01	1.17E+09	1.00	5.00E-02	37.0	3.84	55.45	Bishop & Salisbury (1955)
Chordata	Mammalia	*Bos taurus*	1.39E-01	1.45E+09	1.00	5.00E-02	37.0	3.84	72.55	Bishop & Salisbury (1955)
Chordata	Mammalia	*Bos taurus*	1.18E-02	1.00E+08	0.25	6.00E-04	37.0	3.78	129.52	Fernández & Córdoba (2014)
Chordata	Mammalia	*Bos taurus*	6.14E-02	8.30E+08	1.00	1.50E-02	37.0	3.84	106.63	Murdoch & White (1966)
Chordata	Mammalia	*Bos taurus*	6.75E-02	7.50E+08	1.00	1.50E-02	37.0	3.84	117.19	Murdoch & White (1966)
Chordata	Mammalia	*Bos taurus*	4.92E-02	1.23E+09	3.00	3.00E-03	37.0	3.84	1281.2<br>5	Scott et al (1962)
Chordata	Mammalia	*Bos taurus*	6.31E-03	5.00E+07	0.03	3.00E-04	39.0	3.72	18.85	Garrett et al. (2008)
Chordata	Mammalia	*Bos taurus*	8.72E-03	5.00E+07	0.03	3.00E-04	39.0	3.72	26.04	Garrett et al. (2008)
Chordata	Mammalia	*Bos taurus*	8.90E-03	5.00E+07	0.03	3.00E-04	39.0	3.72	26.58	Garrett et al. (2008)
Chordata	Mammalia	*Bos taurus*	1.78E-02	5.00E+07	0.03	3.00E-04	39.0	3.72	53.07	Garrett et al. (2008)
Chordata	Mammalia	*Canis familiaris*	7.18E-03	1.75E+08	1.00	6.00E-03	37.0	3.84	31.14	Murdoch & White (1966)
Chordata	Mammalia	*Canis familiaris*	1.66E-02	2.55E+08	1.00	6.00E-03	37.0	3.84	71.94	Murdoch & White (1966)
Chordata	Mammalia	*Cavia porcellus*	5.02E-02	1.50E+08	0.50	1.50E-03	37.0	3.84	435.45	Rogers et al. (1979)
Chordata	Mammalia	*Homo sapiens*	1.38E-03	6.14E+06	0.17	4.00E-05	37.0	3.84	150.27	Deutch et al. (1985)
Chordata	Mammalia	*Homo sapiens*	1.69E-03	1.50E+07	0.17	4.00E-05	37.0	3.84	183.66	Deutch et al. (1985)
Chordata	Mammalia	*Homo sapiens*	1.77E-03	8.90E+06	0.17	4.00E-05	37.0	3.84	192.55	Deutch et al. (1985)
Chordata	Mammalia	*Homo sapiens*	2.02E-03	2.17E+07	0.17	4.00E-05	37.0	3.84	219.13	Deutch et al. (1985)
Chordata	Mammalia	*Homo sapiens*	2.94E-03	8.32E+07	0.17	4.00E-05	37.0	3.84	318.93	Deutch et al. (1985)
Chordata	Mammalia	*Homo sapiens*	3.12E-03	6.83E+07	0.17	4.00E-05	37.0	3.84	338.66	Deutch et al. (1985)
Chordata	Mammalia	*Homo sapiens*	1.13E-03	2.00E+07	0.25	2.00E-03	37.0	3.84	3.68	Le Foll et al (2021)
Chordata	Mammalia	*Macropus eugenii*	1.17E-02	1.00E+08	1.00	5.00E-03	34.0	4.05	57.53	Murdoch et al. (1999b)
Chordata	Mammalia	*Macropus eugenii*	1.20E-02	1.00E+08	1.00	5.00E-03	34.0	4.05	59.01	Murdoch et al. (1999b)
Chordata	Mammalia	*Macropus eugenii*	1.62E-02	1.00E+08	1.00	5.00E-03	34.0	4.05	80.00	Murdoch et al. (1999b)
Chordata	Mammalia	*Macropus eugenii*	3.38E-02	1.30E+08	1.00	5.00E-03	37.0	3.84	176.04	Murdoch and Jones (1998)
Chordata	Mammalia	*Mus musculus*	7.13E-06	1.20E+05	2.00	1.20E-05	37.0	3.84	30.95	Boell & Burkus (1984)
Chordata	Mammalia	*Mus musculus*	1.29E-05	1.48E+05	1.50	1.20E-05	37.0	3.84	42.02	Boell (1985)
Chordata	Mammalia	*Mus musculus*	2.93E-05	7.40E+04	1.50	1.20E-05	37.0	3.84	95.41	Boell (1985)

Journal of Experimental Biology • Supplementary information

Chordata	Mammalia	*Mus musculus*	8.35E-05	3.37E+05	3.00	1.20E-05	37.0	3.84	543.83	Boell (1985)
Chordata	Mammalia	*Mus musculus*	4.23E-04	2.00E+06	0.43	5.00E-04	37.0	3.84	9.56	Tourmente et al (2015)
Chordata	Mammalia	*Mus spicilegus*	7.02E-04	2.00E+06	0.43	5.00E-04	37.0	3.84	15.84	Tourmente et al (2015)
Chordata	Mammalia	*Mus spretus*	5.52E-04	2.00E+06	0.43	5.00E-04	37.0	3.84	12.46	Tourmente et al (2015)
Chordata	Mammalia	*Oryctolagus cuniculus*	2.43E-02	7.40E+07	0.33	3.00E-03	37.0	3.84	70.25	Foley & Williams (1967)
Chordata	Mammalia	*Oryctolagus cuniculus*	1.16E-02	1.70E+08	1.00	6.00E-03	37.0	3.84	50.17	Hamner & Williams (1963)
Chordata	Mammalia	*Oryctolagus cuniculus*	6.12E-03	3.40E+08	1.00	6.00E-03	37.0	3.84	26.56	Murdoch & White (1966)
Chordata	Mammalia	*Oryctolagus cuniculus*	1.32E-02	4.10E+08	1.00	6.00E-03	37.0	3.84	57.29	Murdoch & White (1966)
Chordata	Mammalia	*Ovis aries*	1.14E-01	1.06E+09	1.00	2.20E-02	37.0	3.84	135.01	Amir & Schindler (1967)
Chordata	Mammalia	*Ovis aries*	2.43E-01	2.10E+09	1.00	2.20E-02	37.0	3.84	287.95	Amir & Schindler (1967)
Chordata	Mammalia	*Ovis aries*	4.56E-01	3.80E+09	1.00	2.20E-02	37.0	3.84	539.32	Amir & Schindler (1967)
Chordata	Mammalia	*Ovis aries*	6.54E-01	7.40E+09	1.00	2.20E-02	37.0	3.84	774.34	Amir & Schindler (1967)
Chordata	Mammalia	*Ovis aries*	5.16E-02	1.00E+08	0.50	7.00E-03	37.0	3.84	95.98	Black et al. (1968)
Chordata	Mammalia	*Ovis aries*	2.99E-02	9.00E+07	0.33	3.00E-03	37.0	3.84	86.41	Foley & Williams (1967)
Chordata	Mammalia	*Ovis aries*	9.44E-02	7.80E+08	1.00	1.50E-02	37.0	3.84	163.85	Murdoch & White (1966)
Chordata	Mammalia	*Ovis aries*	1.28E-01	1.00E+09	1.00	1.50E-02	37.0	3.84	222.22	Murdoch & White (1966)
Chordata	Mammalia	*Ovis aries*	2.96E-02	2.00E+08	0.50	6.00E-03	37.0	3.84	64.24	Voglmayr et al. (1970)
Chordata	Mammalia	*Ovis aries*	4.72E-02	2.00E+08	0.50	6.00E-03	37.0	3.84	102.43	Voglmayr et al. (1970)
Chordata	Mammalia	*Rattus norvegicus*	6.92E-03	7.50E+07	1.00	6.00E-03	34.0	4.05	28.46	Murdoch et al. (1999a)
Chordata	Mammalia	*Rattus norvegicus*	4.70E-04	1.00E+07	0.17	1.00E-03	36.0	3.91	2.01	Yamashiro et al (2010)
Chordata	Mammalia	*Rattus norvegicus*	2.96E-03	2.00E+06	0.17	2.00E-03	37.0	3.84	6.42	Mitra and Chowdury (1992)
Chordata	Mammalia	*Sus scrofa*	4.39E-03	2.40E+07	0.33	3.00E-03	38.0	3.78	12.90	Foley & Williams (1967)
Echinodermata	Echinoidae	*Anthocidaris crassispina*	4.82E-03	1.00E+08	0.25	3.00E-03	20.0	5.21	7.72	Fujiwara et al. (1982)
Echinodermata	Echinoidae	*Anthocidaris crassispina*	5.09E-03	1.00E+08	0.25	3.00E-03	20.0	5.21	8.15	Fujiwara et al. (1982)
Echinodermata	Echinoidae	*Anthocidaris crassispina*	5.13E-03	1.00E+08	0.25	3.00E-03	20.0	5.21	8.21	Fujiwara et al. (1982)
Echinodermata	Echinoidae	*Clypeaster japonicus*	3.25E-03	1.00E+08	0.25	3.00E-03	20.0	5.21	5.20	Fujiwara et al. (1982)
Echinodermata	Echinoidae	*Clypeaster japonicus*	3.41E-03	1.00E+08	0.25	3.00E-03	20.0	5.21	5.46	Fujiwara et al. (1982)
Echinodermata	Echinoidae	*Clypeaster japonicus*	4.09E-03	1.00E+08	0.25	3.00E-03	20.0	5.21	6.54	Fujiwara et al. (1982)
Echinodermata	Echinoidae	*Echinus esculentus*	5.08E-03	1.12E+09	1.00	1.30E-02	15.0	5.75	6.79	Rothschild & Tuft (1948)
Echinodermata	Echinoidae	*Echinus esculentus*	5.74E-03	6.79E+08	1.00	1.30E-02	15.0	5.75	7.67	Rothschild & Tuft (1948)
Echinodermata	Echinoidae	*Echinus esculentus*	2.83E-03	3.36E+08	2.00	1.50E-02	15.0	5.75	6.55	Rothschild (1950)

Echinodermata	Echinoidae	*Echinus esculentus*	3.93E-03	6.41E+08	6.25	1.50E-02	15.0	5.75	28.45	Rothschild (1950)
Echinodermata	Echinoidae	*Heliocidaris erythrogramma*	3.80E-05	3.75E+05	1.00	7.50E-04	21.0	5.11	0.99	This study (unpub)
Echinodermata	Echinoidae	*Heliocidaris erythrogramma*	8.14E-05	1.29E+06	0.75	7.50E-04	21.0	5.11	1.59	This study (unpub)
Echinodermata	Echinoidae	*Heliocidaris erythrogramma*	1.64E-04	3.00E+06	1.00	7.50E-04	21.0	5.11	4.28	This study (unpub)
Echinodermata	Echinoidae	*Hemicentrotus pulcherrimus*	2.42E-02	1.30E+09	0.07	2.10E-03	17.0	5.53	13.89	Ohtake (1976)
Echinodermata	Echinoidae	*Hemicentrotus pulcherrimus*	5.51E-03	1.00E+08	0.25	3.00E-03	20.0	5.21	8.81	Fujiwara et al. (1982)
Echinodermata	Echinoidae	*Hemicentrotus pulcherrimus*	5.58E-03	1.00E+08	0.25	3.00E-03	20.0	5.21	8.92	Fujiwara et al. (1982)
Echinodermata	Echinoidae	*Hemicentrotus pulcherrimus*	5.58E-03	1.00E+08	0.25	3.00E-03	20.0	5.21	8.92	Fujiwara et al. (1982)
Echinodermata	Echinoidae	*Hemicentrotus pulcherrimus*	3.41E-03	2.00E+08	0.33	3.00E-03	20.0	5.21	7.28	Fujiwara et al. (1983)
Echinodermata	Echinoidae	*Pseudocentrotus depressus*	2.90E-02	1.46E+08	0.07	2.10E-03	18.0	5.42	16.96	Ohtake (1976)
Echinodermata	Echinoidae	*Pseudocentrotus depressus*	5.23E-02	2.60E+08	0.07	2.10E-03	18.0	5.42	30.66	Ohtake (1976)
Echinodermata	Echinoidae	*Pseudocentrotus depressus*	1.11E-01	7.29E+08	0.08	2.10E-03	18.0	5.42	81.55	Ohtake (1976)
Echinodermata	Echinoidae	*Pseudocentrotus depressus*	1.86E-01	1.40E+09	0.08	2.10E-03	18.0	5.42	136.18	Ohtake (1976)
Echinodermata	Echinoidae	*Pseudocentrotus depressus*	3.02E-01	3.16E+09	0.08	2.10E-03	18.0	5.42	220.99	Ohtake (1976)
Echinodermata	Echinoidae	*Pseudocentrotus depressus*	4.70E-03	1.00E+08	0.25	3.00E-03	20.0	5.21	7.52	Fujiwara et al. (1982)
Echinodermata	Echinoidae	*Pseudocentrotus depressus*	5.00E-03	1.00E+08	0.25	3.00E-03	20.0	5.21	8.00	Fujiwara et al. (1982)
Echinodermata	Echinoidae	*Pseudocentrotus depressus*	5.43E-03	1.00E+08	0.25	3.00E-03	20.0	5.21	8.68	Fujiwara et al. (1982)
Echinodermata	Echinoidae	*Pseudocentrotus depressus*	3.05E-02	1.47E+08	0.25	3.00E-03	20.0	5.21	48.78	Kazama and Hino (2012)
Echinodermata	Echinoidae	*Pseudocentrotus depressus*	5.64E-05	1.00E+05	0.05	3.00E-03	20.0	5.21	0.02	Kazama et al (2006)
Echinodermata	Echinoidae	*Pseudocentrotus depressus*	1.02E-02	2.00E+08	0.17	3.00E-03	20.0	5.21	10.89	Yasumasu et al. (1980)
Echinodermata	Echinoidae	*Strongylocentrotus intermedius*	5.74E-03	1.00E+08	0.25	3.00E-03	20.0	5.21	9.18	Fujiwara et al. (1982)
Echinodermata	Echinoidae	*Toxopneustes pileolus*	4.31E-03	1.00E+08	0.25	3.00E-03	20.0	5.21	6.90	Fujiwara et al. (1982)
Echinodermata	Echinoidae	*Toxopneustes pileolus*	4.72E-03	1.00E+08	0.25	3.00E-03	20.0	5.21	7.55	Fujiwara et al. (1982)
Echinodermata	Echinoidae	*Toxopneustes pileolus*	4.81E-03	1.00E+08	0.25	3.00E-03	20.0	5.21	7.70	Fujiwara et al. (1982)

Journal of Experimental Biology • Supplementary information

References:

Ackerman, D.R. & Roussel, J.D. (1971). Citric acid, lactic acid and oxygen metabolism of frozen-thawed semen from four subhuman primate species. *J Reprod Fertil*, 27, 441–443.

Al-Taha, T.J. & Strzezek, J. (1982). Metabolic activity of bull semen as influenced by cryopreservation procedures. *Pakistan J. Agric. Res*, 3, 215–218.

Amir, D. & Schindler, H. (1967). The effect of high sperm concentrations on the rates of respiration and fructolysis by ram spermatozoa. *J Reprod Fertil*, 13, 93–99.

Anand, M., Yadav, S., Kumar, A., Vaswani, S. & Shukla. (2018). Effect of Dilution and Sperm Concentration on Post Thaw Semen Quality in Barbari Buck. *Journal of Animal Research*, 8.

Anilkumar, G., Sudha, K., Anitha, E. & Subramoniam, T. (1996). Aspects of sperm metabolism in the spermatheca of the brachyuran crab Metopograpsus messor. *Journal of Crustacean Biology*, 310–314.

Ashizawa, K., Maeda, S. & Okauchi, K. (1989). The mechanisms of reversible immobilization of flows spermatozoa at body temperature. *J Reprod Fertil*, 86, 271–276.

Ashizawa, K., Tamiya, E., Okauchi, K. & Nishiyama, H. (1985). Prolonged motility of fowl spermatozoa in vitro due to a low molecular weight factor(s) released from cultured embryonic cells. *Anim Reprod Sci*, 9, 181–188.

Austin, C.R., Lutwak-Mann, C. & Mann, T. (1964). Spermatophores and spermatozoa of squid Loligo pealii. *Proceedings of the Royal Society of London. Series B, Containing papers*, 161, 143–152.

Bade, M.L., Nelson, L. & Wiegers, H. (1956). Oxygen uptake, motility and fructolysis of turkey spermatozoa. *J Appl Physiol*, 9, 91–96.

Bernardini, G., Belgiojoso, P. & Camatini, M. (1988). Xenopus spermatozoon: Is there any correlation between motility and oxygen consumption? *Gamete Res*, 21, 403–408.

Bernstein, G.S. (1957). Effect of Ethylenediamine Tetraacetic Acid (Versene) on Motility, Oxygen Consumption, and Anaerobic Fructolysis of Human Sperm.

Billard, R. & Cosson, M.P. (1992). *Some Problems Related to the Assessment of Sperm Motility in Freshwater Fish*. *J Exp Zool*.

Bishop, D.W. (1942). Oxygen consumption of fox sperm. *Biol Bull*, 83, 353–362.

Bishop, M.W. & Salisbury, G.W. (1955). Effect of dilution with saline and phosphate solutions on oxygen uptake of bull semen. *Am J Physiol*, 181, 114–118.

Black, D.L., Crowley, L. v., Duby, R.T. & Spilman, C.H. (1968). Oviduct secretion in the ewe and the effect of oviduct fluid on oxygen uptake by ram spermatozoa in vitro. *J Reprod Fertil*, 15, 127–130.

Boell, E.J. (1985). Oxygen consumption of mouse sperm and its relationship to capacitation. *Journal of Experimental Zoology*, 234, 105–116.

Boell, E.J. & Burkus, J.K. (1984). Oxygen consumption and motility of mouse sperm as affected by oxidizable substrates and oxygen tension. *Carlsberg Res Commun*, 49, 147–154.

Boryshpolets, S., Dzyuba, B. & Drokin, S. (2009). Pre-spawning water temperature affects sperm respiration and reactivation parameters in male carps. *Fish Physiol Biochem*, 35, 661–668.

Brokaw, C.J. & Benedict, B. (1968). Mechanochemical Coupling in Flagella II. Ejects of viscosity and thiourea on metabolism and motility of Ciona spermatozoa. *J Gen Physiol*, 283–299.

Journal of Experimental Biology • Supplementary information

Browne, R.K., Kaurova, S.A., Uteshev, V.K., Shishova, N. v., McGinnity, D., Figiel, C.R., *et al.* (2015). Sperm motility of externally fertilizing fish and amphibians. *Theriogenology*, 83, 1-13.e8.

Burness, G., Casselman, S.J., Schulte-Hostedde, A.I., Moyes, C.D. & Montgomerie, R. (2004). Sperm swimming speed and energetics vary with sperm competition risk in bluegill (Lepomis macrochirus). *Behav Ecol Sociobiol*, 56, 65–70.

Cameron, J.N. (1986). Solubility of O2 and CO2 At Different Temperatures and Salinities (appendix table). *Principles of Physiological Measurement*, 254–259.

Campbell, A.L., Ellis, R.P., Urbina, M.A., Mourabit, S., Galloway, T.S. & Lewis, C. (2017). Impacts of ocean acidification on sperm develop with exposure time for a polychaete with long lived sperm. *Mar Environ Res*, 129, 268–276.

Cardullo, R.A. & Cone, R.A. (1986). Mechanical immobilization of rat sperm does not change their oxygen consumption Rate. *Biol Reprod*, 34, 820–830.

Carter, G.S. (1932). Iodine compounds and fertilisation: VIII. The effects of Thyroxine and Des-Iodo-Thyroxine on the oxygen consumption of the sperm of the rabbit. *J Exp Biol* , 9, 378–388.

Casas, E., Marquínez, A. & Córdoba, M. (2016). Effect of xanthine-xanthine oxidase-catalase system on bovine sperm oxidative metabolism during capacitation induction, 18.

Castellini, C., Ruggeri, S., Mattioli, S., Bernardini, G., Macchioni, L., Moretti, E., *et al.* (2014). Long-term effects of silver nanoparticles on reproductive activity of rabbit buck. *Syst Biol Reprod Med*, 60, 143–150.

Cejko, B.I., Sarosiek, B., Krejszeff, S. & Kowalski, R.K. (2018). Multiple collections of common carp Cyprinus carpio L. semen during the reproductive period and its effects on sperm quality. *Anim Reprod Sci*, 188, 178–188.

Chaudhuri, D. & Wishart, G.J. (1988). Predicting the fertilising ability of avian semen: The development of an objective colourimetric method for assessing the metabolic activity of fowl spermatozoa. *Br Poult Sci*, 29, 837–845.

Cosson, J., Groison, A.L., Suquet, M., Fauvel, C., Dreanno, C. & Billard, R. (2008). Studying sperm motility in marine fish: An overview on the state of the art. *Journal of Applied Ichthyology*, 24, 460–486.

Demoy-Schneider, M., Levêque, A., Schmitt, N., le Pennec, M. & Cosson, J. (2012). Motility activation and metabolism characteristics of spermatozoa of the black-lip-pearl oyster Pinctada margaritifera var: cumingii (Jameson, 1901). *Theriogenology*, 77, 53–64.

Demoy-Schneider, M., Schmitt, N., le Pennec, G., Suquet, M. & Cosson, J. (2018). Quality assessment of cryopreserved black-lip pearl oyster Pinctada margaritifera spermatozoa. *Aquaculture*, 497, 278–286.

Deutch, D.S., Katz, D.F. & Overstreet, J.W. (1985). Increases in human sperm oxygen consumption at low cell concentrations. *Biol Reprod*, 32, 865–871.

Djakiew, D. & Cardullo, R. (1986). Lower temperature of the cauda epididymidis facilitates the storage of sperm by enhancing oxygen availability. *Gamete Res*, 15, 237–245.

Dreanno, C., Cosson, J., Suquet, M., Cibert, C., Fauvel, C., Dorange, G., *et al.* (1999a). Effects of osmolality, morphology perturbations and intracellular nucleotide content during the movement of sea bass (Dicentrarchus labrax) spermatozoa. *J Reprod Fertil*, 116, 113–125.

Dreanno, C., Cosson, J., Suquet, M., Seguin, F., Dorange, G. & Billard, R. (1999b). Nucleotide content, oxydative phosphorylation, morphology, and fertilizing capacity of turbot (Psetta maxima) spermatozoa during the motility period. *Mol Reprod Dev*, 53, 230–243.

Journal of Experimental Biology • Supplementary information

Dziekońska, A., Fraser, L., Koziorowska-Gilun, M., Strzezek, J., Koziorowski, M. & Kordan, W. (2014). Seasonal-dependent variations in metabolic status of spermatozoa and antioxidant enzyme activity in the reproductive tract fluids of wild boar/domestic pig hybrids. *Pol J Vet Sci*, 17, 307–313.
Fang, Y., Zhao, C., Xiang, H., Zhao, X. & Zhong, R. (2020). Melatonin Inhibits Formation of Mitochondrial Permeability Transition Pores and Improves Oxidative Phosphorylation of Frozen-Thawed Ram Sperm. *Front Endocrinol (Lausanne)*, 10.
Fernández, S. & Córdoba, M. (2014). Hyaluronic acid as capacitation inductor: metabolic changes and membrane-associated adenylate cyclase regulation. *Reproduction in Domestic Animals*, 49, 941–946.
Fernández, S. & Córdoba, M. (2016). Progesterone causes metabolic changes involving aminotransferases and creatine kinase in cryopreserved bovine spermatozoa. *Anim Reprod Sci*, 164, 90–96.
Ferramosca, A., Conte, A., Moscatelli, N. & Zara, V. (2016). A high-fat diet negatively affects rat sperm mitochondrial respiration. *Andrology*, 4, 520–525.
Ferramosca, A., Lorenzetti, S., di Giacomo, M., Lunetti, P., Murrieri, F., Capobianco, L., *et al.* (2021). Modulation of Human Sperm Mitochondrial Respiration Efficiency by Plant Polyphenols.
Ferramosca, A., Provenzano, S.P., Coppola, L. & Zara, V. (2012). Mitochondrial respiratory efficiency is positively correlated with human sperm motility. *Urology*, 79, 809–814.
Foley, C.W. & Williams, W.L. (1967). Effect of bicarbonate and oviduct fluid on respiration of spermatozoa. *Proceedings of the Society for Experimental Biology and Medicine*, 126, 634–637.
le Foll, N., Pont, J.C., L'hostis, A., Guilbert, T., Bouillaud, F., Wolf, J.P., *et al.* (2021). Cyclic FEE peptide improves human sperm movement parameters without modification of their energy metabolism. *Int J Mol Sci*, 22.
Frenkel, G.P., Kaplan, R., Homonnai, Z.T. & Kraicer, P.F. (1978). The effect of caffeine on rat epididymal spermatozoa: Motility, metabolism and fertilizing capacity. *Int J Androl*, 1, 416–423.
Froman, D.P., Feltmann, A.J., Rhoads, M.L. & Kirby, J.D. (1999). Sperm mobility: A primary determinant of fertility in the domestic fowl (Gallus domesticus). *Biol Reprod*, 61, 400–405.
Froman, D.P. & Kirby, J.D. (2005). Sperm mobility: Phenotype in roosters (Gallus domesticus) determined by mitochondrial function. *Biol Reprod*, 72, 562–567.
Fujiwara, A., Hino, A., Hiruma, T. & Yasumasu, I. (1982). Inhibition of respiration in sea urchin spermatozoa following interaction with fixed unfertilized eggs VI. Probable difference between the species in the mechanism for the fixed-egg-induced inhibition of sperm respiration. *Dev Growth Differ*, 24, 145–154.
Fujiwara, A., Tazawa, E., Kamata, Y. & Yasumasu, I. (1999). Photo-activation of respiration in degenerated sperm of echiuroid, oyster and sea urchin. *Zoolog Sci*, 16, 237–246.
Fujiwara, A., Yasumasu, I. & Mohri, H. (1983). Change in the respiratory rate of sea urchin spermatozoa following sperm-egg Interaction in the absence of Mg2+. *Dev Growth Differ*, 25, 239–247.
Gallo, A., Esposito, M.C., Tosti, E. & Boni, R. (2021). Sperm motility, oxidative status, and mitochondrial activity: Exploring correlation in different species. *Antioxidants*, 10.
Garrett, L.J.A., Revell, S.G. & Leese, H.J. (2008). Adenosine triphosphate production by bovine spermatozoa and its relationship to semen fertilizing ability. *J Androl*, 29, 449–458.

Journal of Experimental Biology • Supplementary information

Ginsburg, K.A., RandallArmant, D. & Randall Armant, D. (1990). *FERTILITY AND STERILITY The influence of chamber characteristics on the reliability of sperm concentration and movement measurements obtained by manual and videomicrographic analysis**.

Grotjan, H.E., Day, B.N. & Mayer, D.T. (1975). Porcine spermatozoan respiration in the presence of bovine follicular fluid. *J Anim Sci*, 40, 96–98.

Hamner, C.E. & Williams, W.L. (1963). Effect of the female reproductive tract on sperm metabolism in the rabbit and fowl. *Journal of Reproductive Fertilization*, 5, 143–150.

van der Horst, G. (1986). Respiratory physiology of fish, amphibian, and mammalian sperm. *Syst Biol Reprod Med*, 17, 161–164.

Ibrahim, A.A., Hamada, T.A. & Moussa, M.M. (1981). Effect of Varicocele on Sperm Respiration and Metabolism. *Andrologia*, 13, 253–259.

Jacky Cosson, by, Groison, A.-L., suquet, M. & FAuveL, C. (2008). Motility characteristics of spermatozoa in cod (Gadus morhua) and hake (Merluccius merluccius). *Cybium*, 32, 176–177.

Kazama, M., Asami, K. & Hino, A. (2006). Fertilization induced changes in sea urchin sperm: Mitochondrial deformation and phosphatidylserine exposure. *Mol Reprod Dev*, 73, 1303–1311.

Kazama, M. & Hino, A. (2012). Sea urchin spermatozoa generate at least two reactive oxygen species; the type of reactive oxygen species changes under different conditions. *Mol Reprod Dev*, 79, 283–295.

Keogh, L.M., Byrne, P.G. & Silla, A.J. (2018). Effect of long-term dietary beta-carotene supplementation on sperm concentration and motility in an endangered amphibian. *Anim Reprod Sci*, 195, 259–265.

Killian, G.J., Gellerinter, E. & Chapman, D.A. (1985). Alterations of oxygen uptake and the redox state of ubiquinone in rabbit sperm exposed to a variety of physiologic treatments. *Biol Reprod*, 33, 859–869.

Kupriyanova, E.K. & Havenhand, J.N. (2005). Effects of temperature on sperm swimming behaviour, respiration and fertilization success in the serpulid polychaete, Galeolaria caespitosa (Annelida: Serpulidae). *Invertebr Reprod Dev*, 48, 7–17.

Lahnsteiner, F., Berger, B. & Weismann, T. (1999a). Sperm metabolism of the teleost fishes Chalcalburnus chalcoides and Oncorhynchus mykiss and its relation to motility and viability. *Journal of Experimental Zoology*, 284, 454–465.

Lahnsteiner, F., Berger, B. & Weismann, T. (1999b). Sperm metabolism of the teleost fishes Chalcalburnus chalcoides and Oncorhynchus mykiss and its relation to motility and viability. *Journal of Experimental Zoology*, 284, 454–465.

Lahnsteiner, F., Berger, B., Weismann, T. & Patzner, R. (1995). Fine structure and motility of spermatozoa and composition of the seminal plasma in the perch. *J Fish Biol*, 47, 492–508.

Lahnsteiner, F., Berger, R., Weismann, T. & Patzner', R. (1997). Sperm motility and seminal fluid composition in the burbot, Lota lota. *J. Appl. Ichthyol*, 13, 113–119.

Lahnsteiner, F. & Caberlotto, S. (2012). Motility of gilthead seabream Sparus aurata spermatozoa and its relation to temperature, energy metabolism and oxidative stress. *Aquaculture*, 370–371, 76–83.

Lardy, H.A. & Phillips, P.H. (1942). Effect of pH and certain electrolytes on the metabolism of ejaculated spermatozoa. *J Chem Inf Model*, 53, 1689–1699.

Locatello, L., Rigoni, G., Soriano, M.E., Rasotto, M.B. & Poli, F. (2020). Going beyond conventional parameters to unveil sperm quality in fish: The use of fibre optic technology to assess mitochondrial respiratory performance. *Biol Open*, 9, 1–6.

Journal of Experimental Biology • Supplementary information

Magdanz, V., Boryshpolets, S., Ridzewski, C., Eckel, B. & Reinhardt, K. (2019). The motility-based swim-up technique separates bull sperm based on differences in metabolic rates and tail length. *PLoS One*, 14.
Mann, T., Minotakis, C.S. & Polge, C. (1963). Semen composition and metabolism in the stallion and jackass. *J. Reprod. Fertil.*, 5, 109–122.
Mansour, N., Lahnsteiner, F. & Berger, B. (2003). Metabolism of intratesticular spermatozoa of a tropical teleost fish (Clarias gariepinus). *Comparative Biochemistry and Physiology Part B*, 135, 285–296.
Miki, K., Qu, W., Goulding, E.H., Willis, W.D., Bunch, D.O., Strader, L.F., *et al.* (2004). Glyceraldehyde 3-phosphate dehydrogenase-S, a sperm-specific glycolytic enzyme, is required for sperm motility and male fertility. *PNAS*, 101, 16501–16506.
Miró, J., Lobo, V., Quintero-Moreno, A., Medrano, A., Peña, A. & Rigau, T. (2005). Sperm motility patterns and metabolism in Catalonian donkey semen. *Theriogenology*, 63, 1706–1716.
Mita, M., Oguchi, A., Kikuyama, S., Yasumasu, I., de Santis, R. & Nakamura, M. (1994). Endogenous substrates for energy metabolism in spermatozoa of the sea urchins Arbacia lixula and Paracentrotus lividus. *Biological Bulletin*, 186, 285–290.
Mitra, J. & Chowdury, M. (1992). Glycerylphosphorylcholine (GPC) diesterase related alterations in the oxygen consumption profile of rat spermatozoa in differing functional states. *Int J Androl*, 15, 345–354.
Mohri, H. (1956). Studies on the respiration of sea-urchin spermatozoa II. The cytochrome oxidase activity in relation to the dilution effects. *Journal of Experimental Biology*, 33, 330–337.
Murdoch, B.R.N. & White, G. (1968). The influence of the female genital tract on the metabolism of rabbit spermatozoa. *Aust. J. biol. Sci.*, 21, 961–972.
Murdoch, R.N., Armstrong, V.L., Clulow, J. & Jones, R.C. (1999a). Relationship between motility and oxygen consumption of sperm from the cauda epididymides of the rat. *Reprod Fertil Dev*, 11, 87–94.
Murdoch, R.N. & Jones, R.C. (1998). The metabolic properties of spermatozoa from the epididymis of the tammar wallaby, Macropus eugenii. *Mol Reprod Dev*, 49, 92–99.
Murdoch, R.N., Jones, R.C., Wade, M. & Lin, M. (1999b). The ultrastructure and metabolism of ejaculated tammar wallaby sperm are impaired by swim-up procedures when compared with sperm from the cauda epididymidis. *Reprod Fertil Dev*, 11, 263–271.
Murdoch, R.N. & White, I.G. (1966). The metabolism of glucose, fructose, acetate, lactate and pyruvate by ram, bull, dog and rabbit spermatozoa. *J Reprod Fertil*, 12, 271–278.
Nesci, S., Spinaci, M., Galeati, G., Nerozzi, C., Pagliarani, A., Algieri, C., *et al.* (2020). Sperm function and mitochondrial activity: An insight on boar sperm metabolism. *Theriogenology*, 144, 82–88.
Norman, C., Johnson, C.E., Porterfield, I.D. & Dunbar, R.S. (1958). Effect of pH on the Life-Span and Metabolism of Bovine Sperm Kept at Room Temperatures,. *J Dairy Sci*, 41, 1803–1812.
Ohtake, H. (1976). Respiratory behaviour of sea-urchin spermatozoa. I. Effect of pH and egg water on the respiratory rate. *Journal of Experimental Zoology*, 198, 303–311.
Ouzzani, M., Hammady, H., Fedorowicz, Z. & Elmagarmid, A. (2016). Rayyan-a web and mobile app for systematic reviews. *Syst Rev*, 5.
Pacey, A.A. & Bentley, M.G. (1993). Agonists of sperm maturation in Arenicola marina (Annelida: Polychaeta) increase the oxygen consumption of sperm suspensions in vitro. *Invertebr Reprod Dev*, 24, 27–38.

Journal of Experimental Biology • Supplementary information

Rahi, D., Dzyuba, B., Policar, T., Malinovskyi, O., Rodina, M. & Dzyuba, V. (2021). Bioenergetic pathways in the sperm of an under-ice spawning fish, burbot (Lota lota): The role of mitochondrial respiration in a varying thermal environment. *Biology (Basel)*, 10.

Rahi, D., Dzyuba, B., Xin, M., Cheng, Y. & Dzyuba, V. (2020). Energy pathways associated with sustained spermatozoon motility in the endangered Siberian sturgeon Acipenser baerii. *J Fish Biol*, 97, 435–443.

del Rio, A.G. (1979). Metabolism of amphibian spermatozoa in relation to their motility. *Experientia*, 35, 185–186.

Rodger, J.C. & Suter, D.A.I. (1978). Respiration rates and sugar utilization by marsupial spermatozoa. *Gamete Res*, 1, 111–116.

Rogers, B.J., Chang, L. & Yanagimachi, R. (1979). Glucose effect on respiration: Possible mechanism for capacitation in guinea pig spermatozoa. *Journal of Experimental Zoology*, 207, 107–112.

Rothschild, Lord. (1950). The respiration of sea-urchin spermatozoa. *J Exp Biol*, 27, 420–436.

Rothschild, Lord. (1956). The Respiratory Dilution Effect in Sea-urchin Spermatozoa. *Vie et Milieu*, 7, 405–412.

Rothschild, Lord & Tuft, P.H. (1950). The physiology of sea-urchin spermatozoa. The dilution effect in relation to copper and zinc. *J Exp Biol*, 25, 353–368.

Sadeghi, S., Gallego, R. del, García-Colomer, B., Gómez, E.A., Yániz, J.L., Gosálvez, J., *et al.* (2020). Effect of sperm concentration and storage temperature on goat spermatozoa during liquid storage. *Biology (Basel)*, 9, 1–13.

Schindler, H. & Lehrer, A.R. (1968). The Effect of Egg Albumen and of Sperm Immobilization on the Rate of Oxygen Uptake by Fowl Spermatozoa, 36–40.

Schoff, P.K. (1995). Mitochondrial Calcium uptake stimulated by Cibacron Blue F3GA in bovine sperm. *Archives of Biochemistry and Physics*, 318, 349–355.

Schoff, P.K. & First, N.L. (1995). Stimulation of Bovine Sperm Motility and Respiration by the Triazine Dye Cibacron Blue F3GA. *Mol Reprod Dev*, 42, 65–71.

Scott, T.W., White, I.G., Annison ; Geyer, E.F., Marshall, R.P., Ryan, L., Westhaver, M.T., *et al.* (1962). Glucose and acetate metabolism by ram, bull, dog and fowl spermatozoa. *Arch. Biochem. Bio. phys*, 176, 398.

Sexton, T.J. (1974). Oxidative and glycolytic activity of chicken and turkey spermatozoa. *Comp. Bioehem. Physiol*, 48, 59–65.

Simpson, A.M., Swan, M.A. & White, I.G. (1987). Calcium uptake, respiration, and ultrastructure of sperm exposed to ionophore A23187. *Syst Biol Reprod Med*, 19, 5–18.

Stendardi, A., Focarelli, R., Piomboni, P., Palumberi, D., Serafini, F., Ferramosca, A., *et al.* (2011). Evaluation of mitochondrial respiratory efficiency during in vitro capacitation of human spermatozoa. *Int J Androl*, 34, 247–255.

Terner, C. & Korsh, G. (1963). The oxidative metabolism of pyruvate, acetate and glucose in isolated fish spermatozoa. *J Cell Comp Physiol*, 62, 243–249.

Tourmente, M., Sansegundo, E., Rial, E. & Roldan, E.R.S. (2022). Capacitation promotes a shift in the energy metabolism in murine sperm. *bioRxiv*.

Tourmente, M., Villar-Moya, P., Rial, E. & Roldan, E.R.S. (2015). Differences in ATP generation via glycolysis and oxidative phosphorylation and relationships with sperm motility in mouse species. *Journal of Biological Chemistry*, 290, 20613–20626.

Verma, L.R. & Shuel, R.W. (1973). Respiratory metabolism of the semen of the honey-bee, Apis mellifera. *J Insect Physiol*, 19, 97–103.

Journal of Experimental Biology • Supplementary information

Voglmayr, J.K., Murdoch, R.N. & White, I.G. (1970). Metabolism of ram testicular spermatozoa in the presence of testosterone and related steroids. *Acta Endocrinol (Copenh)*, 65, 565–576.
Winchester, C.F. & McKenzie, F.F. (1941). Influence of cell concentration on respiration rate of sperm. *Proceedings of the Society for Experimental Biology and Medicine*, 48, 654–656.
Yamashiro, H., Toyomizu, M., Kikusato, M., Toyama, N., Sugimura, S., Hoshino, Y., *et al.* (2010a). Lactate and Adenosine Triphosphate in the Extender Enhance the Cryosurvival of Rat Epididymal Sperm. *Journal of the American Association for Laboratory Animal Science*, 49, 160–166.
Yamashiro, H., Toyomizu, M., Toyama, N., Aono, N., Sakurai, M., Hiradate, Y., *et al.* (2010b). Extracellular ATP and Dibutyryl cAMP Enhance the Freezability of Rat Epididymal Sperm. *Journal of the American Association for Laboratory Animal Science*, 49, 167–172.
Yasumasu, I., Fujiwara, A. & Hino, A. (1980). Inhibition of respiration in sea urchin spermatozoa following interaction with fixed unfertilized eggs. II. Capacity of the glutaraldehyde fixed unfertilized eggs for the inhibition of the sperm respiration. *Dev Growth Differ*, 22, 421–428.
